# Dammarane-Type Triterpenoid from the Stem Bark of *Aglaia elliptica* (Meliaceae) and Its Cytotoxic Activities

**DOI:** 10.3390/molecules27196757

**Published:** 2022-10-10

**Authors:** Kindi Farabi, Desi Harneti, Darwati, Tri Mayanti, Nurlelasari, Rani Maharani, Aprilia Permata Sari, Tati Herlina, Ace Tatang Hidayat, Unang Supratman, Sofa Fajriah, Mohamad Nurul Azmi, Yoshihito Shiono

**Affiliations:** 1 Department of Chemistry, Faculty of Mathematics and Natural Sciences, Universitas Padjadjaran, Jatinangor 45363, Indonesia; 2 Central Laboratory of Universitas Padjadjaran, Jatinangor 45363, Indonesia; 3 Research Center for Chemistry, National Research and Innovation Agency (BRIN) Kawasan PUSPIPTEK Serpong, South Tangerang 15314, Indonesia; 4 School of Chemical Sciences, Universiti Sains Malaysia, Penang 11800, Malaysia; 5 Department of Food, Life, and Environmental Science, Faculty of Agriculture, Yamagata University, Tsuruoka 997-8555, Japan

**Keywords:** dammarane-type triterpenoid fatty acid ester, *Aglaia elliptica*, cytotoxic activity, MCF-7 cell line, B16-F10 cell line

## Abstract

Two new dammarane-type triterpenoid fatty acid ester derivatives, 3β-oleate-20*S*-hydroxydammar-24-en (**1**) and 3β-oleate-20*S*,24*S*-epoxy-25-hydroxydammarane (**2**) with a known dammarane-type triterpenoid compound, such as 20*S*-hydroxydammar-24-en-3-on (**3**), were isolated from the stem bark of *Aglaia* *elliptica* (C.DC.) Blume. The chemical structures were determined by spectroscopic methods, including FTIR, NMR (one and two-dimensional), and HRESITOF-MS analysis, as well as chemical derivatization and comparison with previous literature. Furthermore, the synthetic analog resulting from transesterification of **1** and **2** also obtained 3β,20*S*-dihydroxy-dammar-24-en (**4**) and 20*S*,24*S*-epoxy-3β,25-dihydroxydammarane (**5**), respectively. The cytotoxic effect of all isolated and synthetic analog compounds was evaluated using PrestoBlue reagent against MCF-7 breast cancer cell and B16-F10 melanoma cell lines. The 20*S*-hydroxydammar-24-en-3-on (**3**) showed the strongest activity against MCF-7 breast cancer and B16-F10 melanoma cell, indicating that the ketone group at C-3 in **3** plays an essential role in the cytotoxicity of dammarane-type triterpenoid. On the other hand, compounds **1** and **2** had very weak cytotoxic activity against the two cell lines, indicating the presence of fatty acid, significantly decreasing cytotoxic activity. This showed the significance of the discovery to investigate the essential structural feature in dammarane-type triterpenoid, specifically for the future development of anticancer drugs.

## 1. Introduction

The *Aglaia* is the largest genus of the Meliaceae family, consisting of 150 species mainly distributed in tropical and sub-tropical regions such as Asia, Northern Australia, and the Pacific. Furthermore, approximately 65 species grow in Indonesia [1,2]. This plant is used traditionally in the country to treat wounds, fever, and skin disease [3]. On the other hand, phytochemical research of *Aglaia* genus revealed a number of diterpenoids [4,5], triterpenoids [6,7], sesquiterpenoids [8,9], limonoids [10,11], steroids [12], flavaglines [13], bisamides [14], and lignans [15,16]. The biological activity of the extracts and secondary metabolites includes cytotoxic [17,18], insecticidal [19], anti-inflammatory [5], antifungal [20], and molluscicide [21].

Dammarane triterpenoid is one of the secondary metabolite groups commonly discovered in the *Aglaia* genus. Approximately 29 dammarane-type triterpenoids have been successively isolated from the *Aglaia* genus [1]. This type of compound showed various bioactivity, such as cytotoxic activity. Zhang et al. [22] isolated six new dammarane triterpenoid compounds that showed potential cytotoxicity against leukemia cancer cell (K562), hepatocellular carcinoma (SMMC-7721), breast (MCF-7), and oral epithelial cancer (KB). Novel dammarane-type triterpenoids isolated from *A. eximia* and *A. smithii* were cytotoxic against P388 murin leukemia cells [6,23]. Oktaviani et al. [24] isolated three dammarane-type triterpenoids and showed cytotoxicity against cervical (HeLa) and human prostate cancer cells (DU145). Therefore, they can be used as an anticancer agent in the future.

*Aglaia elliptica* is a member of the *Aglaia* genus, which is widely grown in Indonesia, specifically on Kalimantan Island [12]. Previous research revealed its potency in producing compounds with cytotoxic activity against cancer cell lines [12,25,26]. There is a need for further exploration, since only 9 dammarane-type triterpenoids have been isolated from this species.

**Figure 1:**
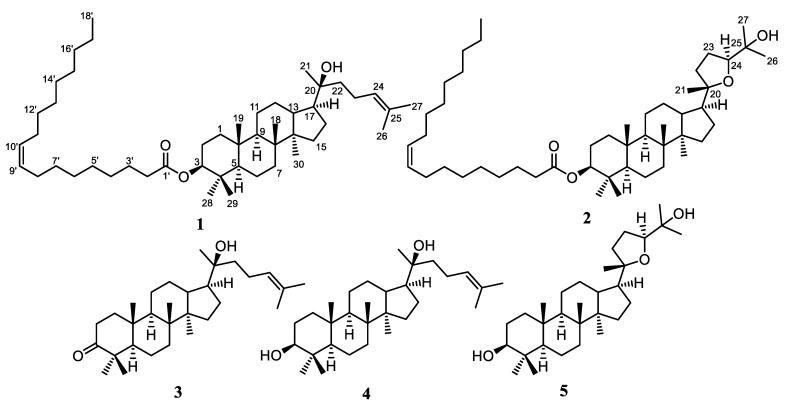
Structure of compounds **1**–**5**.

The isolation and structure elucidation of two new dammarane-type triterpenoid fatty acid esters, namely 3β-oleate-20*S*-hydroxydammar-24-en (**1**) and 3β-oleate-20*S*,24*S*-epoxy-25-hydroxydammarane (**2**), using a variety of chromatographic and spectroscopic technique are described in this research. Furthermore, a known dammarane-type triterpenoid (**3**) and its synthetic analogs were reported through transesterification of **1** and **2** (**4** and **5**, respectively), as shown in Figure 1. The compounds were tested for their cytotoxic activity against MCF-7 breast cancer cells and B16-F10 melanoma cell lines. Therefore, this research briefly explains the structure-activity relationship of **1**–**5** against those cancer cell lines.

## 2. Results and Discussions

### 2.1. Structural Elucidation of the Isolated Compounds

The concentrated methanolic extract of stem bark of *A. elliptica* was dissolved in water and extracted successively with *n*-hexane, ethyl acetate, and *n*-butanol. The *n*-hexane extract has rich triterpenoid content, as shown by the positive Liebermann–Burchard test, resulting in a dark purple color. Therefore, it was subjected to further separation and purification. The separation was conducted with vacuum liquid chromatography (VLC), followed by a combination of column chromatography on silica gel 60 to yield three cytotoxic dammarane-type triterpenoids **1**–**3**, as presented in Figure 1. The structure elucidation of new dammarane-type triterpenoid fatty acid esters **1** and **2** were discussed based on spectroscopic evidence.

**Figure 2:**
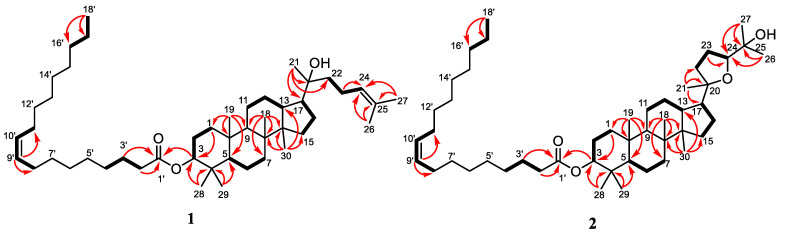
Selected HMBC and ^1^H–^1^H COSY correlations for **1** and **2**.

3β-oleate-20*S*-hydroxydammar-24-en (**1**) was isolated as a colorless oil. The HR-ESI-TOFMS spectra revealed the molecular composition of **1** as C_48_H_84_O_3_ (*m*/*z* 709.6489 [M+H]^+^ calcd. for C_48_H_85_O_3_^+^, *m*/*z* 709.6493), which was established with the NMR data, as shown in Table 1. The IR spectrum indicates the presence of hydroxyl (3490 cm^−1^), a carbonyl ester (1729 cm^−1^), olefinic (1630 cm^−1^), and *gem*-dimethyl groups (1375 cm^−1^). Furthermore, the ^13^C NMR spectrum, with DEPT 135° and assigned by HMQC spectra, revealed the presence of nine methyls, eight methines (including three sp^2^ methines at δ_C_ 124.8, 128.9, and 131.8, and one oxymethine at δ_C_ 80.7), and seven quarternary carbons (including one carbonyl ester at δ_C_ 173.7, one olefinic carbon at δ_C_ 131.7, and one oxygenated quaternary carbon at δ_C_ 75.5). The overlapping signal at δ_C_ 29.2–29.6 indicated the presence of fatty acid substituent in **1**. Three functionalities were observed from seven degrees of unsaturation, and the remaining four degrees accounted for the tetracyclic core of the dammarane-type triterpenoid. The ^1^H-NMR spectrum showed eight tertiary methyls (δ_H_ 0.83, 0.85, 0.85, 0.86, 0.94, 1.12, 1.61, 1.67, each 3H), one primary methyl (δ_H_ 0.85, 3H, t, *J* = 6.5 Hz), three olefinic proton at δ_H_ 5.10 (1H, t, *J* = 7.0 Hz), 5.35 (1H, dd, *J* = 3.5, 9.5 Hz), and 5.40 (1H, dd, *J* = 3.5, 9.5 Hz), as well as one oxymethine proton (δ_H_ 4.47, 1H, dd, *J* = 5.5, 10.5 Hz). A series of overlapping protons at δ_H_ 1.24 with high integral suggests the presence of fatty acid substituent. The comparison of NMR data of **1** with 3β,20*S*-dihydroxy-dammar-24-en isolated from the same species by Hidayat et al. [25] showed high similarity. The primary difference was the presence of fatty acid substituent through ester linkage in **1**. This comparison strengthens the presumption that compound **1** was a fatty acid ester derivative of 3β,20*S*-dihydroxy-dammar-24-en. On the other hand, the established structure was mainly determined by HMBC and ^1^H-^1^H COSY experiments, as shown in Figure 2. The HMBC correlations of each tertiary methyl to the neighboring carbons confirmed the tetracyclic dammarane-type triterpenoid core.

**Figure 3:**
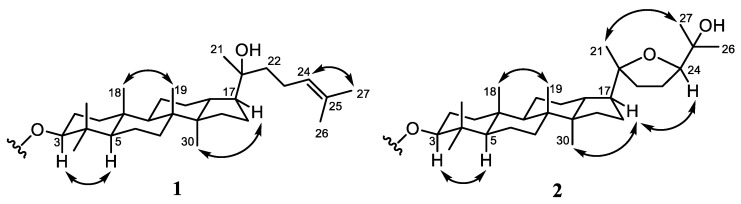
Selected NOESY correlations for **1** and **2**.

The position of the double bond was assigned to be in C-24/C-25 from the correlations of CH_3_-26 (δ_H_ 1.67) and CH_3_-27 (δ_H_ 1.61) to C-24 (δ_C_ 124.8) and C-25 (δ_C_ 131.7) with a ^1^H-^1^H COSY correlation of H-22/H-23/H-24. Furthermore, the hydroxyl group, bounded at C-20, was determined by the correlation of CH_3_-21 (δ_H_ 1.12) to C-20 (δ_C_ 75.5), C-17 (δ_C_ 49.4), and C-22 (δ_C_ 40.6). The primary structural feature that determined the novelty of **1** was determined by the correlation of H-3 (δ_H_ 4.47), H-2’ (δ_H_ 2.27), and H-3’ (δ_H_ 1.59) to ester C-1’ (δ_C_ 173.7), which supported the formation of ester linkage in **1** at C-3, with the confirmation of fatty acid substituent position. Furthermore, the type of fatty acid substituent at dammarane-type triterpenoid was determined by the mass spectrum of the methyl ester product obtained through transesterification reaction [27]. The mass spectrum of the transesterification products of **1** resulted in the identification of methyl oleate [M+H]^+^ *m*/*z* 297.2778 (calcd. for C_19_H_37_O_2_^+^, *m*/*z* 297.2794) and the dammarane-type triterpenoid core moiety corresponds to 3β,20*S*-dihydroxy-dammar-24-en (**4**) based on HR-ESI-TOFMS and NMR data comparison [25]. The HMBC correlation was observed at H-9’ (δ_H_ 5.40) to C-8’ (δ_C_ 32.0) and C-11’ (δ_C_ 32.1), whereas the ^1^H-^1^H COSY cross peak at H-8’/H-9’/H-10′/H-11’ was supported by the presence of oleic acid, which has a double bond at C-9’/C-10’. On the other hand, the relative configuration of **1** was deduced by a NOESY experiment, as shown in Figure 3. The key NOESY correlations observed at H-3/H-5 indicated that the ester group at C-3 was β-oriented. The NOESY cross peak, which was also studied between CH_3_-30/H-17, indicated that the side chain of **1** attached at C-17 was α-oriented. Absolute configuration of the hydroxyl group at C-20 was established to be *S* based on comparing chemical shifts with previously reported analogs [25,28]. Therefore, the structure of **1** as a new dammarane triterpenoid fatty acid ester derivative, 3β-oleate-20*S*-hydroxydammar-24-en, was elucidated.

3β-oleate-20*S*,24*S*-epoxy-25-hydroxydammarane (**2**) was obtained as a colorless oil. The molecular composition of C_48_H_84_O_4_ was established from the HR-ESI-TOFMS spectrum *m*/*z* 725.6444 [M+H]^+^ (calcd. for C_48_H_85_O_4_^+^, *m*/*z* 725.6442) with NMR data, as presented in Table 1. The IR spectrum showed the presence of hydroxyl, a carbonyl ester, olefinic, ether, and gem-dimethyl groups at 3511 cm^−1^, 1731 cm^−1^, 1647 cm^−1^, 1173 cm^−1^, and 1376 cm^−1^, respectively. Furthermore, the ^13^C NMR spectrum detailed by DEPT 135^o^ and HMQC spectra showed the resonances of nine methyls, eight methines (including two sp^2^ methines at δ_C_ 128.9 and 131.7, and two oxymethines at δ_C_ 80.6 and 86.3), and seven quaternary carbons (including one carbonyl ester at δ_C_ 173.6 and two oxygenated quaternary carbon at δ_C_ 70.3 and 86.6). The overlapping carbon signal at approximately δ_C_ 29.2–29.6 indicated the presence of fatty acid substituent in **2**. There are seven degrees of unsaturation, where two observed functionalities and the remaining five degrees were consistent for a tetracyclic dammarane-type triterpenoid core with an epoxide ring in the side chain. The ^1^H-NMR spectrum showed eight tertiary methyls (δ_H_ 0.82, 0.85, 0.87, 0.95, 1.09, 1.12, 1.16, 1.17, each 3H), one primary methyl at δ_H_ 0.85 (3H, t, *J* = 6.5 Hz), two olefinic proton at δ_H_ 5.35 and 5.40 (each 1H, dd, *J* = 3.5, 9.5 Hz), and two oxymethine proton at δ_H_ 3.62 (1H, dd, *J* = 5.5 Hz, 10.5 Hz) and 4.45 (1H, dd, *J* = 5.0, 11.0 Hz). A series of overlapping protons at δ_H_ 1.24 with high integral predicts the fatty acid substituent in **2**. Additionally, a detailed NMR data comparison of **2** with the 20*S*,24*S*-epoxy-3β,25-dihydroxydammarane isolated from the same species [25] showed that the structures of the two compounds are closely related. The main difference was the presence of a fatty acid ester substituent in **2**, and the exact structure of **2** was determined by ^1^H–^1^H COSY and HMBC spectra, as shown in Figure 2.

The tetracyclic dammarane-type triterpenoid core was established based on the correlation of tertiary methyl to the neighboring carbons. The hydroxyl group attached at C-25 was confirmed by the correlation of CH_3_-26 (δ_H_ 1.17) and CH_3_-27 (δ_H_ 1.09) to C-24 (δ_C_ 86.3) and C-25 (δ_C_ 70.3). Furthermore, the formation of tetrahydrofuran or epoxide rings through C-20/C-24 was mainly determined based on the correlation of CH_3_-21 (δ_H_ 1.12) to C-20 (δ_C_ 86.6), C-17 (δ_C_ 49.9), and C-22 (δ_C_ 35.3), as well as H-23 (δ_H_ 1.74) to C-24 (δ_C_ 86.3). The ^1^H-^1^H COSY cross peak of H-22/H-23/H-24 also supported the epoxide ring at C-20/C-24. The ester group between triterpenoid moiety and fatty acid substituent was determined by the correlation of H-3 (δ_H_ 4.45), H-2’ (δ_H_ 2.27), and H-3’ (δ_H_ 1.68) to ester C-1’ (δ_C_ 173.6) through ester linkage in **2** at C-3. Fatty acid substituent was determined using the same methods as compound **1**. Moreover, the mass spectrum resulted in the identification of methyl oleate [M+H]^+^ *m*/*z* 297.2777 (calcd. for C_19_H_37_O_2_^+^, *m*/*z* 297.2794), and the dammarane-type triterpenoid core moiety corresponds to 20*S*,24*S*-epoxy-3β,25-dihydroxydammarane (**5**) based on the comparison of HR-ESI-TOFMS and NMR data [25]. The HMBC correlation was observed at H-9’ (δ_H_ 5.40) to C-8’ (δ_C_ 32.0) and C-11’ (δ_C_ 32.1), whereas the ^1^H-^1^H COSY cross peak at H-8’/H-9’/H-10’/H-11’ was supported by the presence of oleic acid which has a double bond at C-9’/C-10’. According to Figure 3, the relative configuration of **2** was deduced by a NOESY experiment, and the correlations observed at H-3/H-5 indicated that the ester group at C-3 was β-oriented. The NOESY cross peak, also observed between CH_3_-30/H-17, indicated that the side chain of **1** attached at C-17 was α-oriented. The absolute configuration of C-20 and C-24 was established to be the 20*S* and 24*S* based on the chemical shift of epoxy dammarane triterpenoid analogs [21,28]. Consequently, compound **2** was selected as 3β-oleate-20*S*,24*S*-epoxy-25-hydroxydammarane, a new dammarane-type triterpenoid fatty acid ester.

20*S*-hydroxydammar-24-en-3-on (**3**) was obtained as a white amorphous powder. ^1^H-NMR (CDCl_3_, 500 MHz) δ_H_ 1.86 (2H, m, H-1), 2.40 (2H, m, H-2), 1.32 (1H, m, H-5), 1.40 & 1.49 (each 1H, m, H-6), 1.26 & 1.56 (each 1H, m, H-7), 1.36 (1H, m, H-9), 1.25 & 1.45 (each 1H, m, H-11), 1.23 & 1.79 (each 1H, m, H-12), 1.69 (1H, m, H-13), 1.03 & 1.40 (each 1H, m, H-15), 1.44 & 1.69 (each 1H, m, H-16), 1.68 (1H, m, H-17), 0.93 (3H, s, H-18), 0.88 (3H, s, H-19), 1.08 (3H, s, H-21), 1.42 (2H, m, H-22), 1.99 (2H, m, H-23), 5.05 (1H, t, *J* = 5.0 Hz, H-24), 1.62 (3H, s, H-26), 1.56 (3H, s, H-27), 1.01 (3H, s, H-28), 0.97 (3H, s, H-29), 0.82 (3H, s, H-30). ^13^C-NMR (CDCl_3_, 125 MHz) δ_C_ 39.9 (C-1), 34.1 (C-2), 218.0 (C-3), 47.4 (C-4), 55.4 (C-5), 19.7 (C-6), 34.6 (C-7), 40.3 (C-8), 50.0 (C-9), 36.8 (C-10), 22.0 (C-11), 27.5 (C-12), 42.4 (C-13), 50.3 (C-14), 31.2 (C-15), 24.8 (C-16), 49.8 (C-17), 15.2 (C-18), 16.0 (C-19), 75.4 (C-20), 25.5 (C-21), 40.5 (C-22), 22.6 (C-23), 124.8 (C-24), 131.6 (C-25), 25.7 (C-26), 17.7 (C-27), 26.7 (C-28), 21.0 (C-29), 16.4 (C-30). HR-TOFMS *m*/*z* 443.3882 [M+H]^+^ (calcd. for C_30_H_51_O_2_^+^, *m*/*z* 443.3884). Compound **3** had a comparable HR-TOFMS result and chemical shift to 20S-hydroxydammar-24-en-3-on [29]. Therefore, it was identified as 20S-hydroxydammar-24-en-3-on, isolated for the first time from this species.

3β,20*S*-dihydroxy-dammar-24-en (**4**) was obtained as a colorless crystal. ^1^H-NMR (CDCl_3_, 500 MHz) δ_H_ 1.37 & 1.40 (each 1H, m, H-1), 1.43 (2H, m, H-2), 3.37 (1H, t, *J* = 4.5 Hz, H-3), 1.23 (1H, m, H-5), 1.38 (2H, m, H-6), 1.24 & 1.55 (each 1H, m, H-7), 1.42 (1H, m, H-9), 1.52 (2H, m, H-11), 1.53 & 1.91 (each 1H, m, H-12), 1.58 (1H, m, H-13), 1.04 & 1.45 (each 1H, m, H-15), 1.77 (2H, m, H-16), 1.69 (1H, m, H-17), 0.93 (3H, s, H-18), 0.82 (3H, s, H-19), 1.13 (3H, s, H-21), 1.44 (2H, m, H-22), 2.02 (2H, m, H-23), 5.10 (1H, t, *J* = 5.4 Hz, H-24), 1.66 (3H, s, H-26), 1.59 (3H, s, H-27), 0.91 (3H, s, H-28), 0.81 (3H, s, H-29), 0.86 (3H, s, H-30). ^13^C-NMR (CDCl_3_, 125 MHz) δ_C_ 33.7 (C-1), 24.9 (C-2), 76.4 (C-3), 37.7 (C-4), 49.6 (C-5), 18.3 (C-6), 35.2 (C-7), 40.7 (C-8), 50.4 (C-9), 37.3 (C-10), 21.4 (C-11), 25.4 (C-12), 42.3 (C-13), 50.5 (C-14), 31.2 (C-15), 27.6 (C-16), 49.8 (C-17), 15.6 (C-18), 16.1 (C-19), 75.5 (C-20), 25.5 (C-21), 40.6 (C-22), 22.6 (C-23), 124.8 (C-24), 131.7 (C-25), 25.9 (C-26), 17.8 (C-27), 28.4 (C-28), 22.2 (C-29), 16.6 (C-30). HR-TOFMS *m*/*z* 445.4035 [M+H]^+^ (calcd. for C_30_H_53_O_2_^+^, *m*/*z* 445.4040). The HR-TOFMS and chemical shift of synthetic analog 4 were similar with 3β,20*S*-dihydroxy-dammar-24-en [25]. Therefore, compound **4** was identified as 3β,20*S*-dihydroxy-dammar-24-en.

The 20*S*,24*S*-epoxy-3β,25-dihydroxydammarane (**5**) were obtained as a colorless crystal. ^1^H-NMR (CDCl_3_, 500 MHz) δ_H_ 1.42 (2H, m, H-1), 1.55 (2H, m, H-2), 3.38 (1H, t, *J* = 3.0 Hz, H-3), 1.24 (1H, m, H-5), 1.39 (2H, m, H-6), 1.63 (2H, m, H-7), 1.44 (1H, m, H-9), 1.25 & 1.53 (each 1H, m, H-11), 1.23 & 1.75 (each 1H, m, H-12), 1.62 (1H, m, H-13), 1.04 & 1.40 (each 1H, m, H-15), 1.44 & 1.51 (each 1H, m, H-16), 1.44 (1H, m, H-17), 0.84 (3H, s, H-18), 0.87 (3H, s, H-19), 1.13 (3H, s, H-21), 1.22 (2H, m, H-22), 1.85 (2H, m, H-23), 3.62 (1H, dd, *J* = 4.8, 10.2 Hz, H-24), 1.17 (3H, s, H-26), 1.09 (3H, s, H-27), 0.92 (3H, s, H-28), 0.82 (3H, s, H-29), 0.95 (3H, s, H-30). ^13^C-NMR (CDCl_3_, 125 MHz) δ_C_ 33.7 (C-1), 25.4 (C-2), 76.4 (C-3), 37.3 (C-4), 49.6 (C-5), 18.3 (C-6), 34.8 (C-7), 40.7 (C-8), 50.7 (C-9), 37.7 (C-10), 21.7 (C-11), 27.1 (C-12), 42.8 (C-13), 50.2 (C-14), 31.5 (C-15), 25.9 (C-16), 49.8 (C-17), 16.2 (C-18), 16.6 (C-19), 86.7 (C-20), 27.3 (C-21), 35.3 (C-22), 26.4 (C-23), 86.3 (C-24), 70.3 (C-25), 27.9 (C-26), 24.1 (C-27), 28.4 (C-28), 22.2 (C-29), 15.6 (C-30). HR-TOFMS *m*/*z* 461.3993 [M+H]^+^ (calcd. for C_30_H_53_O_3_^+^, *m*/*z* 461.3989). The HR-TOFMS and chemical shift of synthetic analog 5 were similar to the 20*S*,24*S*-epoxy-3β,25-dihydroxydammarane [25]. Therefore, compound **5** was identified as the 20*S*,24*S*-epoxy-3β,25-dihydroxydammarane.

### 2.2. Cytotoxic Activity of All Obtained Compounds

The cytotoxic activity of compounds **1**–**5** was evaluated against MCF-7 breast cancer cells and B16-F10 melanoma cell lines. Cisplatin was used as a positive control, as shown in Table 2. The experimental result and the statistical graphs of IC_50_ are presented in Figures S19–S28.

The cytotoxic activity against MCF-7 breast cancer and B16-F10 melanoma cells of **1** and **2** was very weak compared with the positive control. This indicates that the fatty acid substituent decreased the cytotoxic activity. Bradley et al. [30] reported that the presence of fatty acid substituents could reduce the toxicity of anticancer drugs. Therefore, this observation was appropriate for the literature.

A literature review on dammarane-type triterpenoid, protopanaxadiol, and protopanaxatriol derivatives with no epoxide ring at the side chain showed high anticancer activity in a previous Chinese patent [31]. This implies that the formation of the epoxide ring decreases anticancer activity. Protopanaxadiol and protopanaxatriol derivatives have similar structural features to compound 3 but contain additional hydroxyl groups in C-12, significantly increasing cytotoxic activity. Many structural features influence cytotoxic activity. Based on the observation of the structure-activity relationship, it can be concluded that the absence of cyclization at the side chain and the presence of the ketone group at C-3, for example, in **3** significantly increases cytotoxic activity for both MCF-7 and B16-F10 cells, indicating that **3** has the highest activity compared to others.

## 3. Materials and Methods

### 3.1. General Experimental Procedures

Optical rotations were measured using an ATAGO AP-300 automatic polarimeter (Saitama, Japan), and the high-resolution mass spectra (HRESI-TOFMS) were obtained on a Waters Xevo Q-TOF direct probe/MS system using ESI^+^ mode and microchannel plates MCPs detector (Milford, MA, USA). In contrast, the IR spectra were measured on a One Perkin Elmer infrared-100 (Shelton, CT, USA). The NMR data were recorded on a JEOL ECZ-500 spectrometer (Tokyo, Japan) at 500 MHz for ^1^H and 125 MHz for ^13^C using TMS as the internal standard. Chromatographic separations were conducted on a silica gel G60 (Merck, Darmstadt, Germany, 70–230 and 230–400 mesh), and the TLC plates were precoated with GF_254_ (Merck, 0.25 mm), after which detection was performed by spraying with 10% H_2_SO_4_ in ethanol, before heating.

### 3.2. Plant Material

The stem bark of *A. elliptica* was obtained from the Bogor Botanical Garden, West Java, Indonesia, in June 2015. Subsequently, the plant was identified and classified by the staff of Herbarium Bogoriense, and a voucher specimen with No. Bo-1294562 was deposited at the herbarium.

### 3.3. Extraction and Isolation

The dried stem bark of *A. elliptica* (2.3 kg) was extracted with methanol (12 L) at room temperature for 5 days. Methanol was used to extract nearly all components from plants because of its ability as a magic solvent. After solvent evaporation, 321.5 g extract was obtained, and partition of methanol extract resulted in 22.6 g, 31.4 g, and 34.5 g of the *n*-hexane, ethyl acetate, and *n*-butanol extract, respectively.

A total of 22.6 g of the *n*-hexane extract was fractionated by vacuum liquid chromatography (r: 5 cm; h: 12 cm) on silica gel (300 g of G60 silica gel) using a gradient elution of *n*-hexane-ethyl acetate (10:0–0:10, stepwise 10%; v: 500 mL) followed by ethyl acetate-methanol (10:0–0:10, stepwise 10%; v: 500 mL) to yield five fractions (A–E). Subsequently, 6 g of C was separated with silica gel column chromatography (70–230 mesh, 120 g) using a gradient elution of *n*-hexane-ethyl acetate (10:0–1:1 stepwise 5%; r: 4 cm; h: 15 cm; v: 400 mL) to yield five fractions (C1–C5). A total of 2.1 g of C3 was separated with silica gel column chromatography (70–230 mesh, 63 g) using a gradient elution of *n*-hexane-ethyl acetate (10:0–7:3 stepwise 2.5%; r: 2 cm; h: 15 cm; v: 240 mL) to produce eight fractions (C3a-C3h). Approximately 300 mg of C3b fraction was separated with silica gel column chromatography (230–400 mesh, 18 g) using *n*-hexane: methylene chloride and ethyl acetate in a ratio of 20:2:1 to yield six fractions (C3b1-C3b6). Moreover, C3b2 produced compound **1** (7 mg), and 73 mg of C3b4 fraction was then separated with silica gel column chromatography (230–400 mesh, 2.8 g) using *n*-hexane-ethyl acetate in a ratio of 20:1 to produce C3b4a and C3b4b. C3b4a also produced compound **2** (8.8 mg), and 516 mg of C3c was separated with silica gel column chromatography (230–400 mesh, 21 g) using *n*-hexane-ethyl acetate in a ratio of 23:2 to yield three fractions, such as C3c1-C3c3. Finally, compound **3** (5 mg) was produced by recrystallizing 50 mg of C3c2.

3β-oleate-20*S*-hydroxydammar-24-en (**1**): colourless oil; [α]^25^_D_ +11.3 (c 0.1, MeOH); IR (KBr) ν_max_ 3490, 2947, 2854, 1729, 1630, 1456, 1375, 1172 cm^−1^; HR-TOFMS *m*/*z* 709.6489 [M+H]^+^ (calcd. for C_48_H_85_O_3_^+^, *m*/*z* 709.6493); ^1^H-NMR (CDCl_3_, 500 MHz) and ^13^C-NMR (CDCl_3_, 125 MHz) shown in Table 1.

3β-oleate-20*S*,24*S*-epoxy-25-hydroxydammarane (**2**): colourless oil; [α]^25^_D_ +15.7 (c 0.1, MeOH); IR (KBr) ν_max_ 3511, 2955, 2853, 1731, 1647, 1454, 1376, 1173 cm^−1^; HR-TOFMS *m*/*z* 725.6444 [M+H]^+^ (calcd. for C_48_H_85_O_4_^+^, *m*/*z* 725.6442); ^1^H-NMR (CDCl_3_, 500 MHz) and ^13^C-NMR (CDCl_3_, 125 MHz) shown in Table 1.

### 3.4. Transesterification of Triterpenoids Fatty Acid Ester

The transesterification of fatty acid triterpene esters **1** and **2** was conducted according to the previously reported procedures [27]. The fatty acid esters at 4 mg each were stirred for 5 h with 0.9 mg sodium methoxide in dry MeOH at 0.5 mL using a magnetic stirrer. Subsequently, the reaction product was extracted with H_2_O and CHCl_3_, and the organic phase was separated, dried with Na_2_SO_4,_ and then concentrated under vacuum to obtain methyl ester of the fatty acid moiety. HCl (1%) was added to the remaining aqueous phase, followed by extraction with CHCl_3_ to yield triterpenoid alcohol moiety crude. Finally, using silica gel column chromatography with *n*-hexane-ethyl acetate in a ratio of 4:1, the triterpenoid moiety crude was purified to yield triterpenoid moiety each **4** (1 mg) and **5** (1.2 mg).

The mass spectrum recorded of the transesterification product of methyl ester **1** and **2** showed [M+H]^+^ *m*/*z* 297.2778 (calcd. for C_19_H_37_O_2_^+^, *m*/*z* 297.2794) and [M+H]^+^ *m*/*z* 297.2777 (calcd. for C_19_H_37_O_2_^+^, *m*/*z* 297.2794), respectively. This research’s Results and Discussion section contains the mass spectrum of triterpenoid alcohol moiety of transesterification products **4** and **5,** along with NMR data.

### 3.5. Determination of Cytotoxic Activity

The cytotoxic bioassay was conducted using the PrestoBlue assay, as reported by Izdihar et al. [32]. Presto Blue reagent (Thermo Fisher Scientific, Uppsala, Sweden) was used to quickly and quantitatively analyze the proliferation of different resazurin-based cell types employing live-cell reduction capabilities. Cells maintain a reduced cytosolic environment when they are alive and healthy. Reducing resazurin (blue) works as a cell viability indicator by using absorbance or fluorescent outputs to decrease resorufin (purple). The conversion is proportional to the number of metabolically active cells. Furthermore, MCF-7 and B16-F10 cell lines grown in 70% confluent were harvested, counted, and diluted with a complete culture RPMI medium. The cells were then transferred into 96-well plates with 170,000 cells/well. They were treated with increasing concentrations of compounds **1–5** (3.91, 7.81, 15.63, 31.25, 62.50, 125, 250, 5000 μg/mL) with co-solvent 2% (*v*/*v*) DMSO in PBS after overnight growth. Cisplatin was used as the positive control, and the samples were incubated at 37 °C in a 5% CO_2_ incubator for 24 h. After incubation, the medium was replaced immediately by a 10 μL PrestoBlue reagent in a 90 μL RPMI medium. The plates were incubated for 1–2 h until resorufin was formed (color changes from blue to purple). The absorbance was measured at 570 nm using a microplate reader. Additionally, the IC_50_ value is the concentration for 50% growth inhibition. The percentage of cytotoxicity compared to untreated cells was determined with the equation below. A plot of % cytotoxicity versus sample concentrations showed 50% cytotoxicity (IC_50_). Finally, all assays and analyses were performed in duplicate and averaged.

## 4. Conclusions

Three dammarane-type triterpenoids, including 3β-oleate-20*S*-hydroxydammar-24-en (**1**), 3β-oleate-20*S*,24*S*-epoxy-25-hydroxydammarane (**2**), and 20*S*-hydroxydammar-24-en-3-on (**3**), were successfully isolated from the stem bark of *Aglaia* *elliptica* (C.DC.) Blume. Compound **1** and **2** were elucidated as new dammarane-type triterpenoids fatty acid ester derivatives, whereas **3** was identified as a known compound. The transesterification of **1** and **2** yielded 3β,20*S*-dihydroxy-dammar-24-en (**4**) and 20*S*,24*S*-epoxy-3β,25-dihydroxydammarane (**5**). Furthermore, **4** and **5** were identified as known compounds. Using PrestoBlue reagent, compounds (1–5) were tested against MCF-7 breast cancer cell and B16-F10 melanoma cell lines. The results showed that compound **3** had the strongest activity against both cell lines. The presence of fatty acid substituent in **1** and **2** decreased cytotoxic activity, whereas the ketone group in **3** significantly increased the activity against both cell lines. Finally, the formation of a tetrahydrofuran ring appears to reduce the cytotoxic activity against both cell lines.

## Figures and Tables

**Table 1:** NMR Data (500 MHz for ^1^H and 125 MHz for ^13^C, in CDCl_3_) for **1** and **2**.

No.	1		2	
	^13^C NMR δc (Mult.)	^1^H NMR δ_H_ (Integral, Mult., *J* = Hz)	^13^C NMR δc (Mult.)	^1^H NMR δ_H_ (Integral, Mult., *J* = Hz)
1	32.7 (t)	1.95 (2H, m)	33.0 (t)	2.21 (2H, m)
2	24.9 (t)	1.48 (2H, m)	23.8 (t)	1.68 (2H, m)
3	80.7 (d)	4.47 (1H, dd, 5.5, 10.5)	80.6 (d)	4.45 (1H, dd, 5.0, 11.0)
4	38.0 (s)	-	37.1 (s)	-
5	56.0 (d)	0.82 (1H, m)	56.0 (d)	0.82 (1H, m)
6	18.2 (t)	1.41 (2H, m)	18.2 (t)	1.51, 1.38 (each 1H, m)
7	35.2 (t)	1.22, 1.54 (each 1H, m)	34.8 (t)	1.65 (1H, m)
8	40.4 (s)	-	40.5 (s)	-
9	50.6 (d)	1.32 (1H, m)	50.8 (d)	1.32 (1H, m)
10	37.1 (s)	-	38.0 (s)	-
11	21.6 (t)	1.48 (2H, m)	21.1 (t)	1.51 (2H, m)
12	25.1 (t)	1.43 (2H, m)	27.0 (t)	1.76 (2H, m)
13	42.3 (d)	1.59 (1H, m)	42.9 (d)	1.61 (1H, m)
14	50.4 (s)	-	50.1 (s)	-
15	31.2 (t)	1.05 (2H, m)	31.5 (t)	1.04 (2H, m)
16	27.6 (t)	1.78 (2H, m)	25.9 (t)	1.25 (2H, m)
17	49.9 (d)	1.71 (1H, m)	49.9 (d)	1.85 (1H, m)
18	15.6 (q)	0.94 (3H, s)	16.4 (q)	0.85 (3H, s)
19	16.3 (q)	0.85 (3H, s)	16.6 (q)	0.87 (3H, s)
20	75.5 (s)	-	86.6 (s)	-
21	25.5 (q)	1.12 (3H, s)	27.2 (q)	1.12 (3H, s)
22	40.6 (t)	1.45 (2H, m)	35.3 (t)	1.26 (2H, m)
23	22.7 (t)	1.28 (2H, m)	26.4 (t)	1.74 (2H, m)
24	124.8 (d)	5.10 (1H, t, 7.0)	86.3 (d)	3.62 (1H, dd, 5.5, 10.0)
25	131.7 (s)	-	70.3 (s)	-
26	25.8 (q)	1.67 (3H, s)	27.9 (q)	1.17 (3H, s)
27	17.8 (q)	1.61 (3H, s)	24.1 (q)	1.09 (3H, s)
28	28.1 (q)	0.83 (3H, s)	28.1 (q)	0.82 (3H, s)
29	16.5 (q)	0.85 (3H, s)	17.5 (q)	1.16 (3H, s)
30	16.6 (q)	0.86 (3H, s)	15.6 (q)	0.95 (3H, s)
1’	173.7 (s)	-	173.6 (s)	-
2’	34.2 (t)	2.27 (2H, t, 7.5)	34.2 (t)	2.27 (2H, t, 7.5)
3’	23.8 (t)	1.59 (2H, m)	23.8 (t)	1.68 (2H, m)
4’	29.2 (t)	1.24 (2H, m)	29.2 (t)	1.24 (2H, m)
5’	29.6 (t)	1.24 (2H, m)	29.6 (t)	1.24 (2H, m)
6’	29.4 (t)	1.24 (2H, m)	29.3 (t)	1.24 (2H, m)
7’	29.7 (t)	1.24 (2H, m)	29.7 (t)	1.24 (2H, m)
8’	32.0 (t)	1.99 (2H, m)	32.0 (t)	2.00 (2H, m)
9’	128.9 (d)	5.40 (1H, dd, 3.5, 9.5)	128.9 (d)	5.40 (1H, dd, 3.5, 10.0)
10’	131.8 (d)	5.35 (1H, dd, 3.5, 9.5)	131.7 (d)	5.35 (1H, dd, 3.5, 10.0)
11’	32.1 (t)	1.99 (2H, m)	32.1 (t)	2.00 (2H, m)
12’	29.4 (t)	1.24 (2H, m)	29.3 (t)	1.24 (2H, m)
13’	29.6 (t)	1.24 (2H, m)	29.6 (t)	1.24 (2H, m)
14’	29.2 (t)	1.24 (2H, m)	29.2 (t)	1.24 (2H, m)
15’	29.4 (t)	1.24 (2H, m)	29.3 (t)	1.24 (2H, m)
16’	29.7 (t)	1.24 (2H, m)	29.7 (t)	1.24 (2H, m)
17’	22.6 (t)	1.22 (2H, m)	22.7 (t)	1.22 (2H, m)
18’	14.2 (q)	0.85 (3H, t, 6.5)	14.2 (q)	0.85 (3H, t, 6.5)

**Table 2:** Cytotoxic activities against MCF-7 and B16F10 cell lines for **1**–**5**.

Compounds	IC_50_ for MCF-7 (μg/mL)	IC_50_ for B16F10 (μg/mL)
3β-oleate-20*S*-hydroxydammar-24-en (**1**)	313.23	181.34
20S,24*S*-epoxy-3β-oleate-25-hydroxydammarane (**2**)	212.21	98.40
20*S*-hydroxydammar-24-en-3-on (**3**)	67.30	22.95
3β,20*S*-dihydroxydammar-24-en (**4**)	121.01	49.57
20*S*,24*S*-epoxy-3β,25-dihydroxydammarane (**5**)	82.61	95.27
Cisplatin (positive control)	53.00	43.00

## Data Availability

All the data in this research were presented in manuscript and Supplementary Material.

## Supplementary Materials

The following supporting information can be downloaded at: https://www.mdpi.com/article/10.3390/molecules27196757/s1, Figure S1: HRTOFMS Spectrum of 1; Figure S2. FTIR Spectrum of 1; Figure S3. ^1^H-NMR Spectrum of 1 (500 MHz in CDCl_3_); Figure S4. ^13^C-NMR Spectrum of 1 (125 MHz in CDCl_3_); Figure S5. DEPT-135° Spectrum of 1 (125 MHz in CDCl_3_); Figure S6. HMQC Spectrum of 1; Figure S7. HMBC Spectrum of 1; Figure S8. ^1^H-^1^H-COSY Spectrum of 1; Figure S9. NOESY Spectrum of 1; Figure S10. HRTOFMS Spectrum of 2; Figure S11. FTIR Spectrum of 2; Figure S12. ^1^H-NMR Spectrum of 2 (500 MHz in CDCl_3_); Figure S13. ^13^C-NMR Spectrum of 2 (125 MHz in CDCl_3_); Figure S14. DEPT-135° Spectrum of 2 (125 MHz in CDCl_3_); Figure S15. HMQC Spectrum of 2; Figure S16. HMBC Spectrum of 2; Figure S17. ^1^H-^1^H-COSY Spectrum of 2; Figure S18. NOESY Spectrum of 2; Figure S19. Results of cytotoxic activity of 1 against MCF-7 cell line; Figure S20. Results of cytotoxic activity of 2 against MCF-7 cell line; Figure S21. Results of cytotoxic activity of 3 against MCF-7 cell line; Figure S22. Results of cytotoxic activity of 4 against MCF-7 cell line; Figure S23. Results of cytotoxic activity of 5 against MCF-7 cell line; Figure S24. Results of cytotoxic activity of 1 against B16-F10 cell line; Figure S25. Results of cytotoxic activity of 2 against B16-F10 cell line; Figure S26. Results of cytotoxic activity of 3 against B16-F10 cell line; Figure S27. Results of cytotoxic activity of 4 against B16-F10 cell line; Figure S28. Results of cytotoxic activity of 5 against B16-F10 cell line.

## Sample Availability

Samples of the compounds **3** and **4** are available from the authors.
