# Supplementary material for: Dammarane-Type Triterpenoid from the Stem Bark of Aglaia elliptica (Meliaceae) and Its Cytotoxic Activities
Source: Molecules. 2022 Oct 10;27(19):6757. doi: 10.3390/molecules27196757 (PMC9571388; doi:10.3390/molecules27196757)

Supplementary Material

Dammarane-Type Triterpenoids from the Stem Bark of *Aglaia elliptica* (Meliaceae) and Their Cytotoxic Activities

Kindi Farabi [1], Desi Harneti [1], Darwati Darwati [1], Tri Mayanti [1], Nurlelasari Nurlelasari [1], Rani Maharani [1,2], Aprilia Permata Sari[1] , Tati Herlina[1] , Ace Tatang Hidayat[1], Unang Supratman [1,2,*], Sofa Fajriah [3], Mohamad Nurul Azmi [4], Yoshihito Shiono [5]

Affiliation

[1]Department of Chemistry, Faculty of Mathematics and Natural Sciences, Universitas Padjadjaran, Jatinangor, 45363, Indonesia

[2]Central Laboratory of Universitas Padjadjaran, Jatinangor, 45363, Indonesia

[3]Research Center for Chemistry, National Research and Innovation Agency (BRIN) Kawasan PUSPIPTEK Serpong Tangeran Selatan, 15314, Banten, Indonesia

[4]School of Chemical Sciences, Universiti Sains Malaysia, 11800 Minden, Penang, Malaysia

[5]Department of Food, Life, and Environmental Science, Faculty of Agriculture, Yamagata University, Tsuruoka, Yamagata, 997-8555, Japan

*Correspondence: unang.supratman@unpad.ac.id; Tel.: +62 22 7794391

Contents

Figure S1. HRTOFMS Spectrum of **1**.

Figure S2. FTIR Spectrum of **1**.

Figure S3. ^{1}H-NMR Spectrum of **1** (500 MHz in $CDCl_3$).

Figure S4. ^{13}C-NMR Spectrum of **1** (125 MHz in $CDCl_3$).

Figure S5. DEPT-135° Spectrum of **1** (125 MHz in $CDCl_3$).

Figure S6. HMQC Spectrum of **1**.

Figure S7. HMBC Spectrum of **1**.

Figure S8. ^{1}H-^{1}H-COSY Spectrum of **1**.

Figure S9. NOESY Spectrum of **1**.

Figure S10. HRTOFMS Spectrum of **2**.

Figure S11. FTIR Spectrum of **2**.

Figure S12. ^{1}H-NMR Spectrum of **2** (500 MHz in $CDCl_3$).

Figure S13. ^{13}C-NMR Spectrum of **2** (125 MHz in $CDCl_3$).

Figure S14. DEPT-135° Spectrum of **2** (125 MHz in $CDCl_3$).

Figure S15. HMQC Spectrum of **2**.

Figure S16. HMBC Spectrum of **2**.

Figure S17. ^{1}H-^{1}H-COSY Spectrum of **2**.

Figure S18. NOESY Spectrum of **2**.

Figure S19. Results of cytotoxic activity of **1** against MCF-7 cell line.

Figure S20. Results of cytotoxic activity of **2** against MCF-7 cell line.

Figure S21. Results of cytotoxic activity of **3** against MCF-7 cell line.

Figure S22. Results of cytotoxic activity of **4** against MCF-7 cell line.

Figure S23. Results of cytotoxic activity of **5** against MCF-7 cell line.

Figure S24. Results of cytotoxic activity of **1** against B16-F10 cell line.

Figure S25. Results of cytotoxic activity of **2** against B16-F10 cell line.

Figure S26. Results of cytotoxic activity of **3** against B16-F10 cell line.

Figure S27. Results of cytotoxic activity of **4** against B16-F10 cell line.

Figure S28. Results of cytotoxic activity of **5** against B16-F10 cell line.

Figure S1. HRTOFMS Spectrum of **1**.

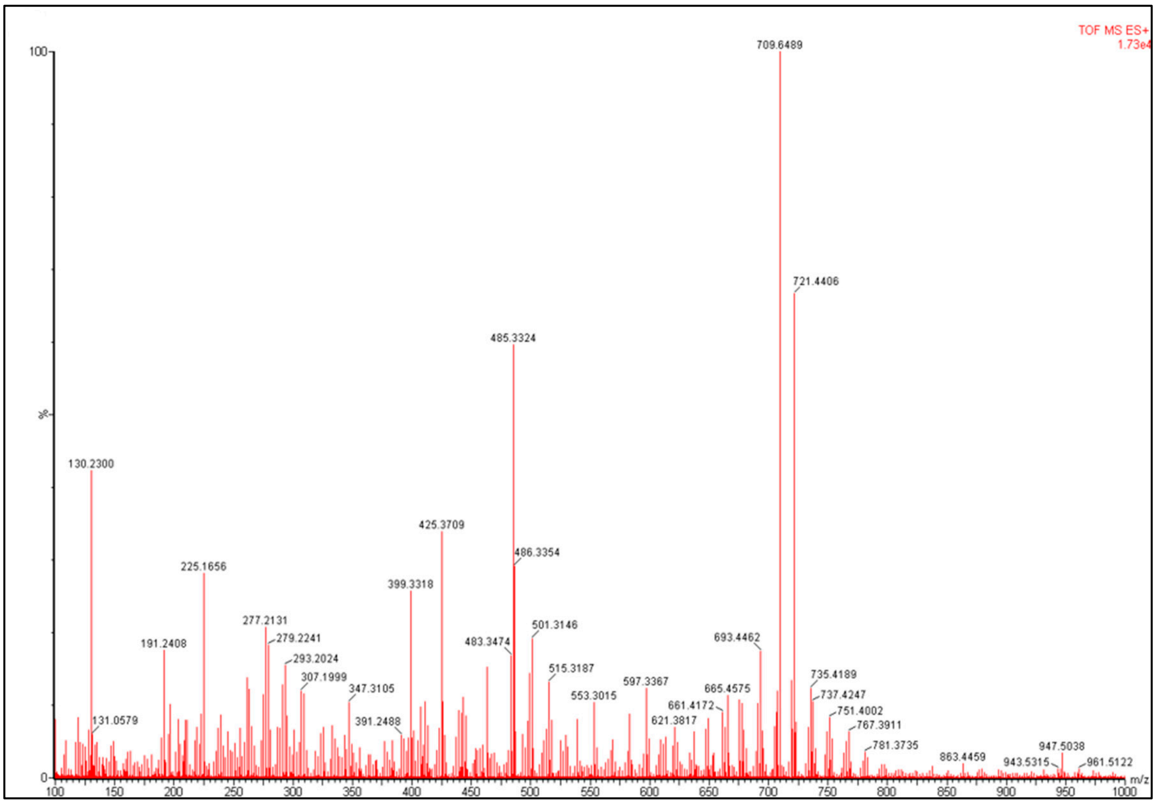

Figure S2. FTIR Spectrum of **1**.

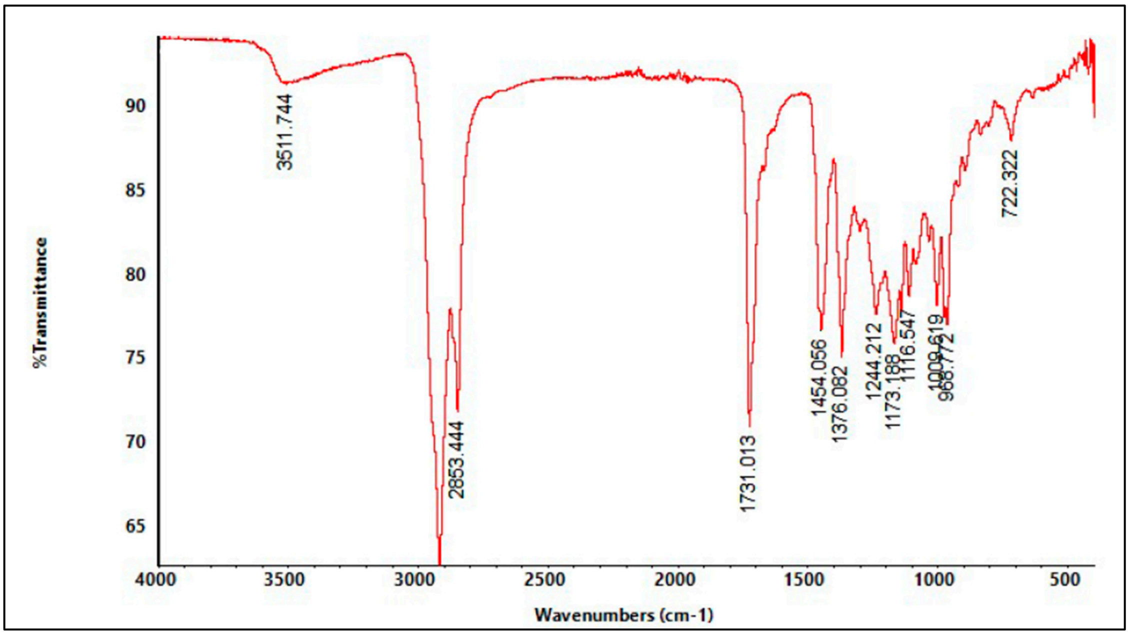

Figure S3. ^{1}H-NMR Spectrum of **1** (500 MHz in $CDCl_3$).

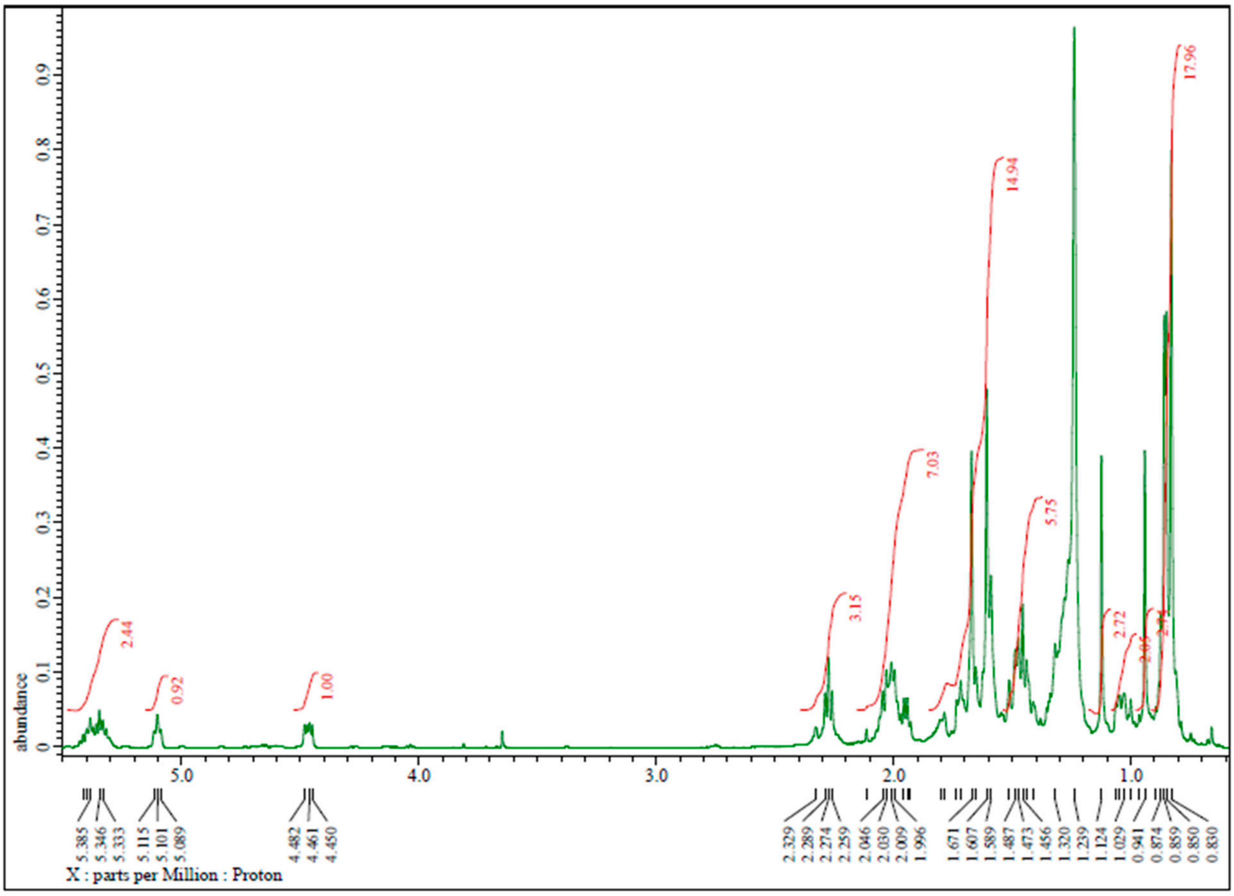

Figure S4. ^{13}C-NMR Spectrum of **1** (125 MHz in $CDCl_3$).

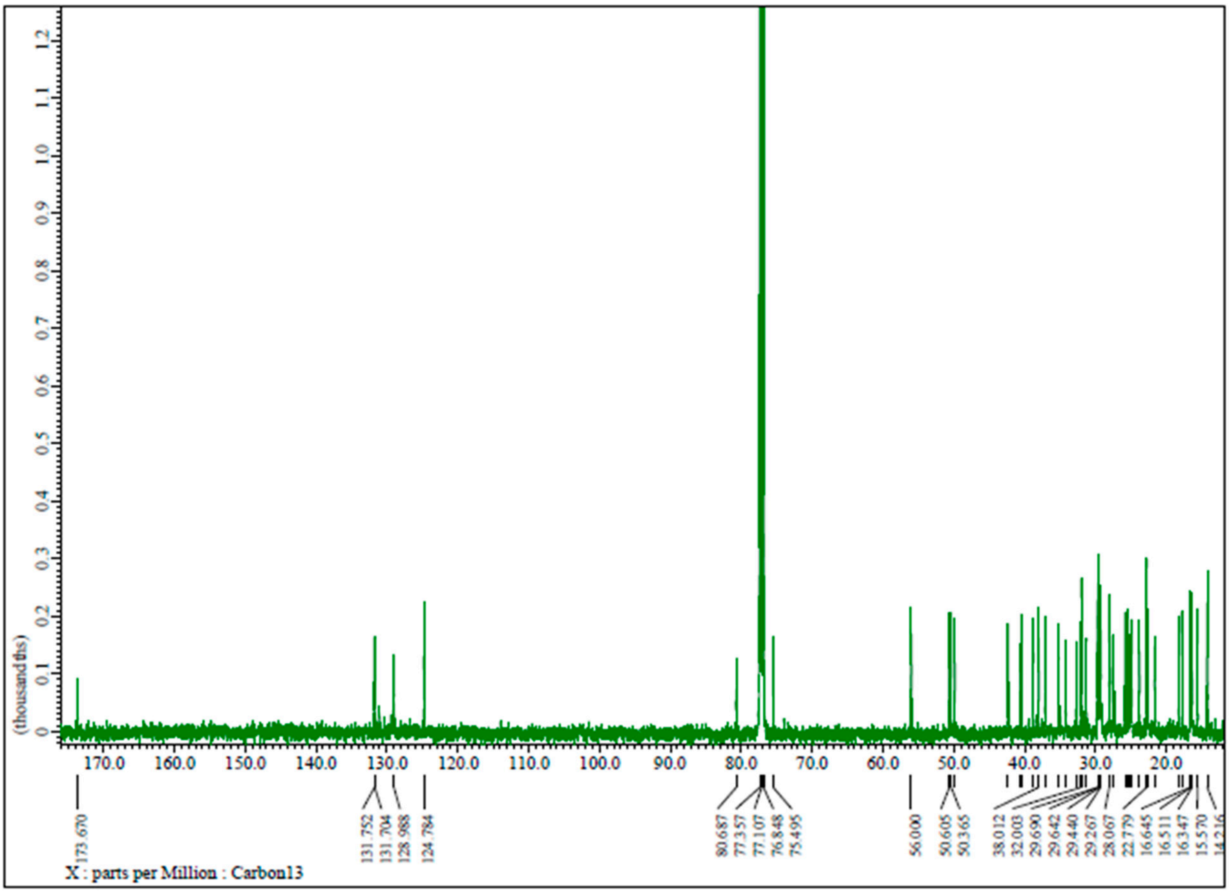

Figure S5. DEPT-135° Spectrum of **1** (125 MHz in $CDCl_3$).

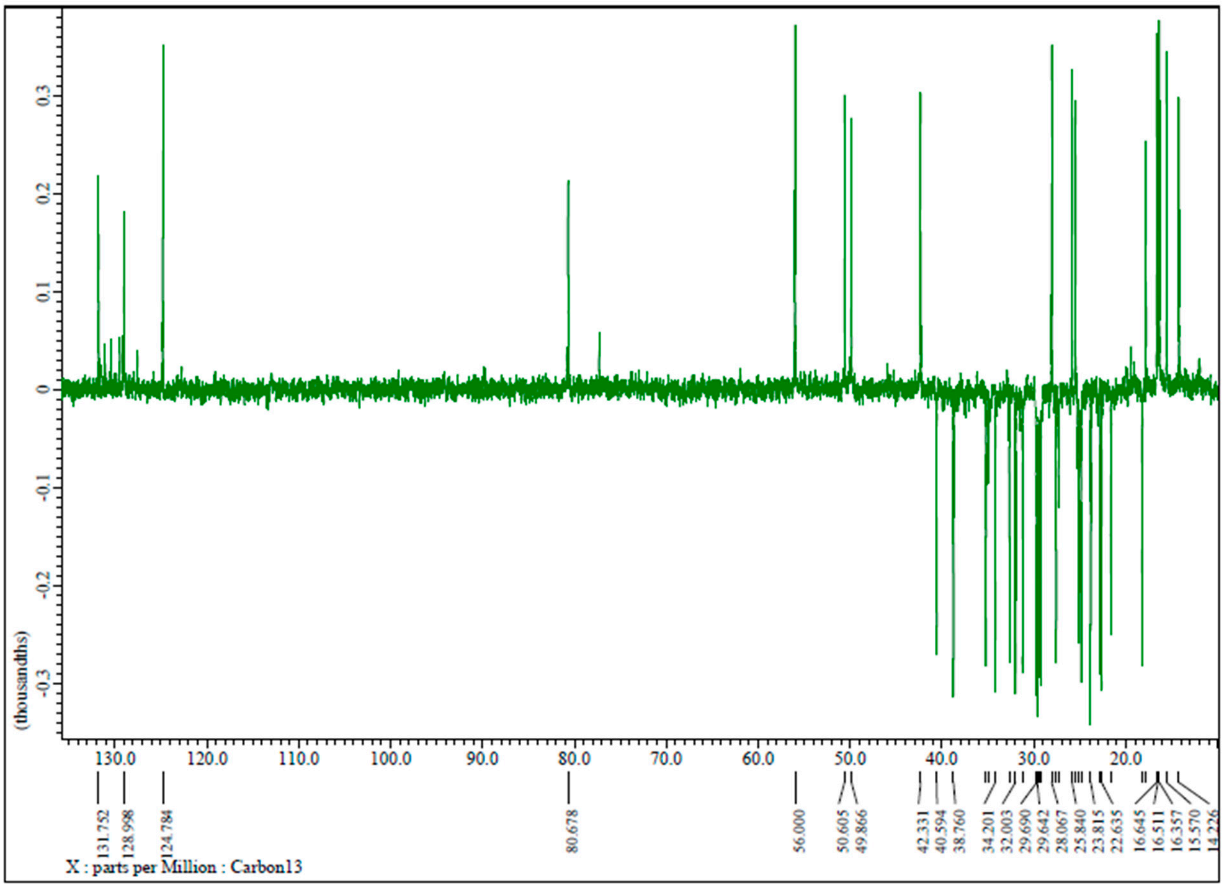

Figure S6. HMQC Spectrum of **1**.

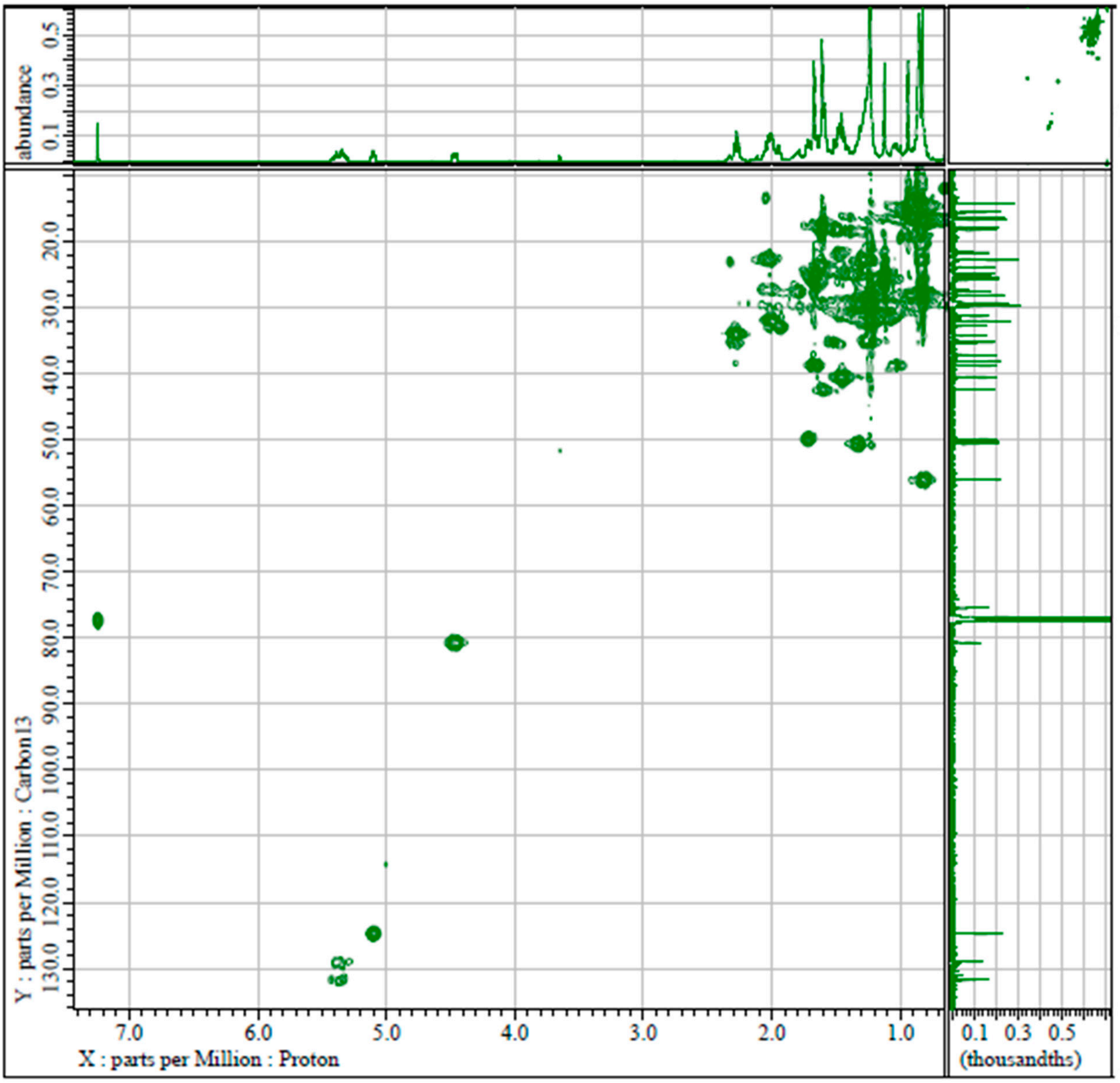

Figure S7. HMBC Spectrum of **1**.

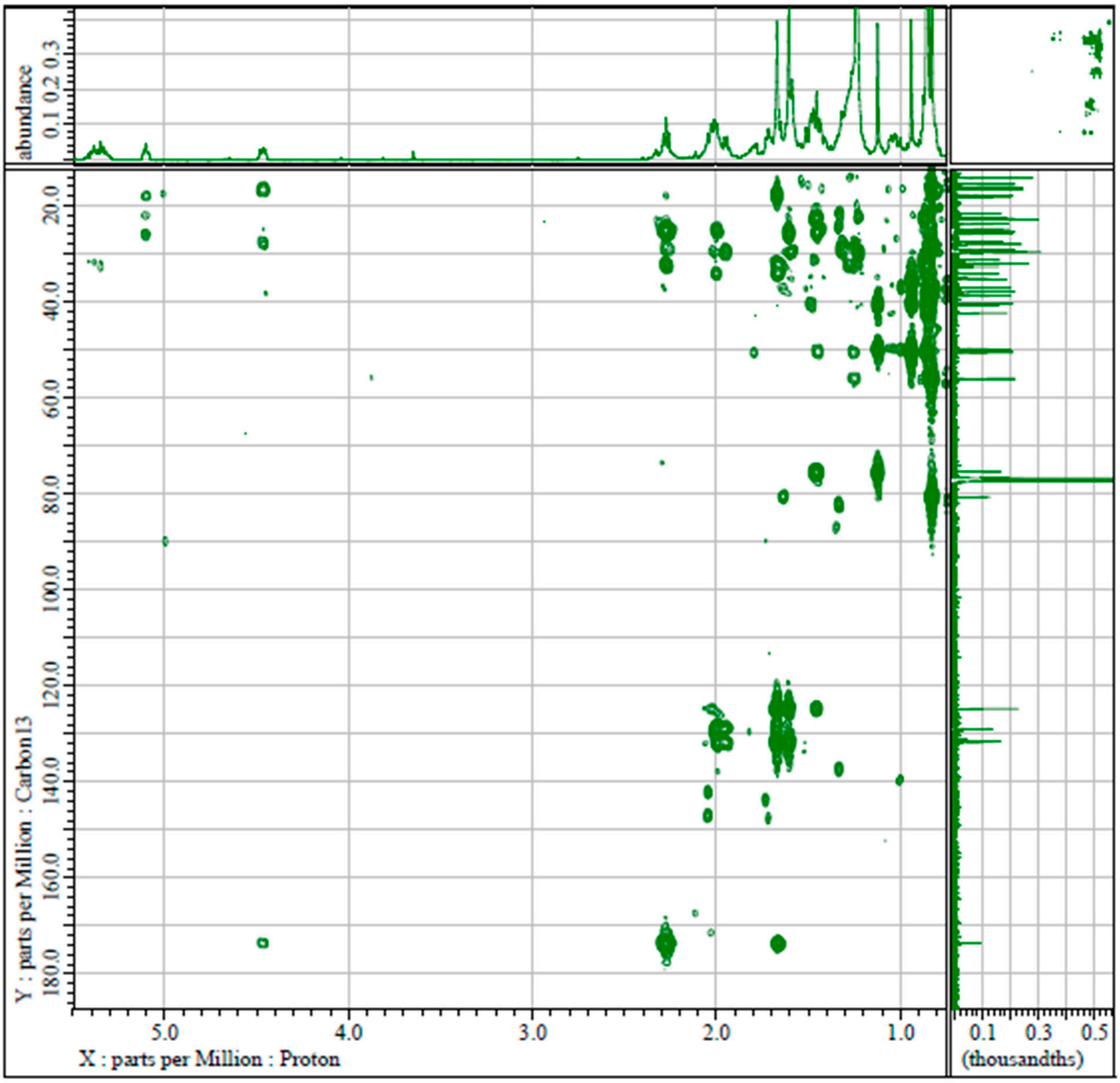

Figure S8. ^1H-^1H-COSY Spectrum of **1**.

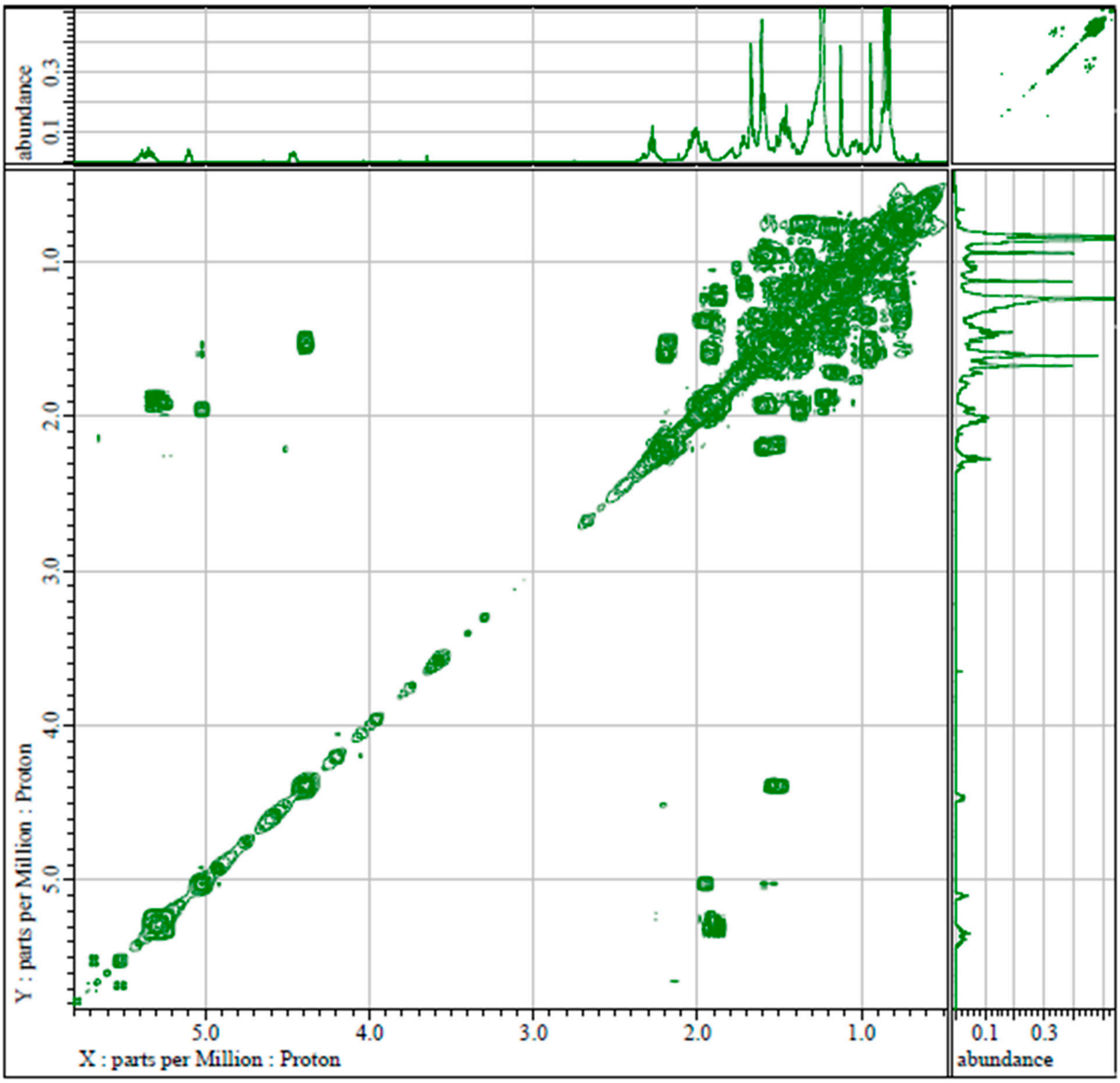

Figure S9. NOESY Spectrum of **1**.

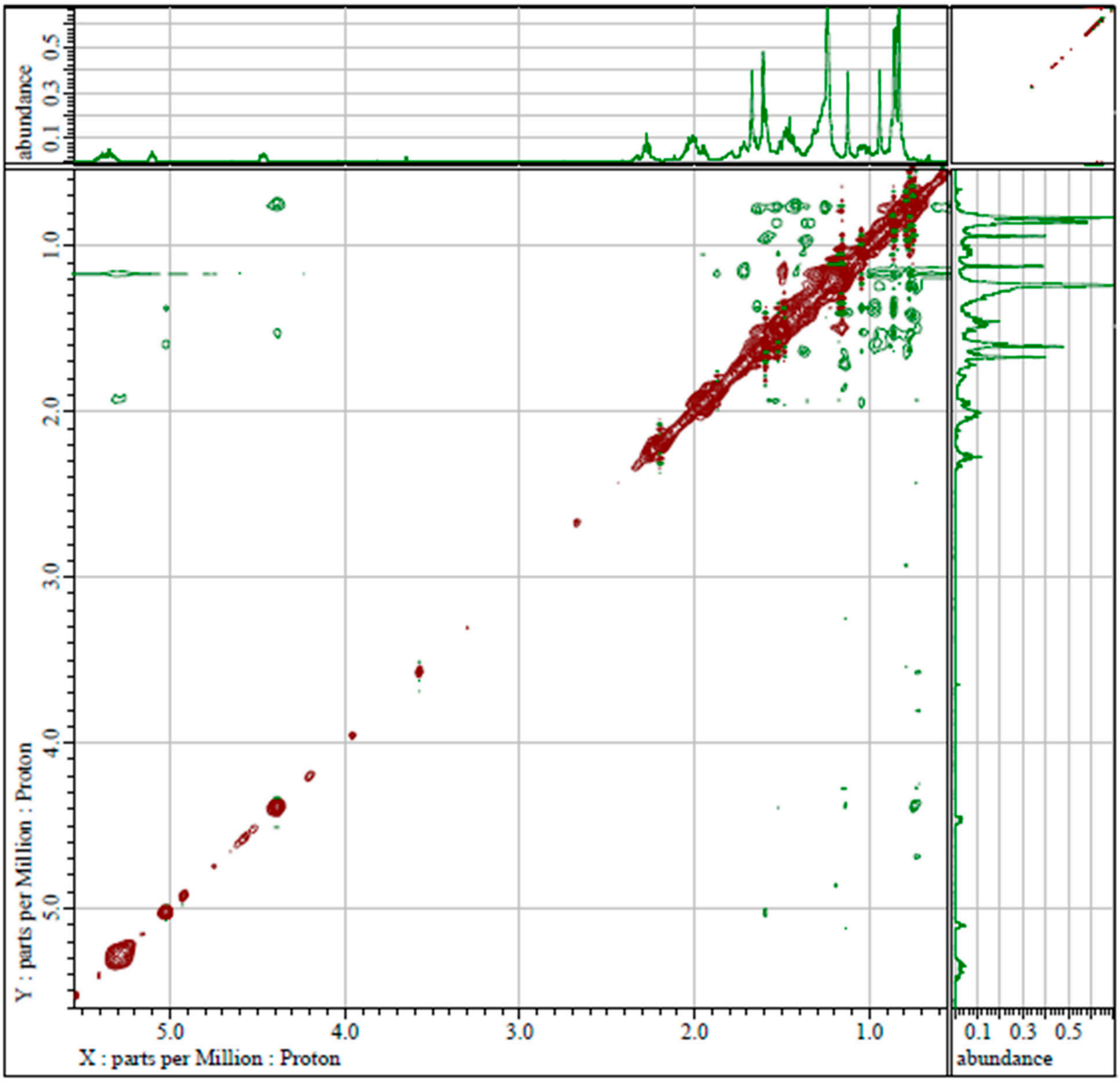

Figure S10. HRTOFMS Spectrum of **2**.

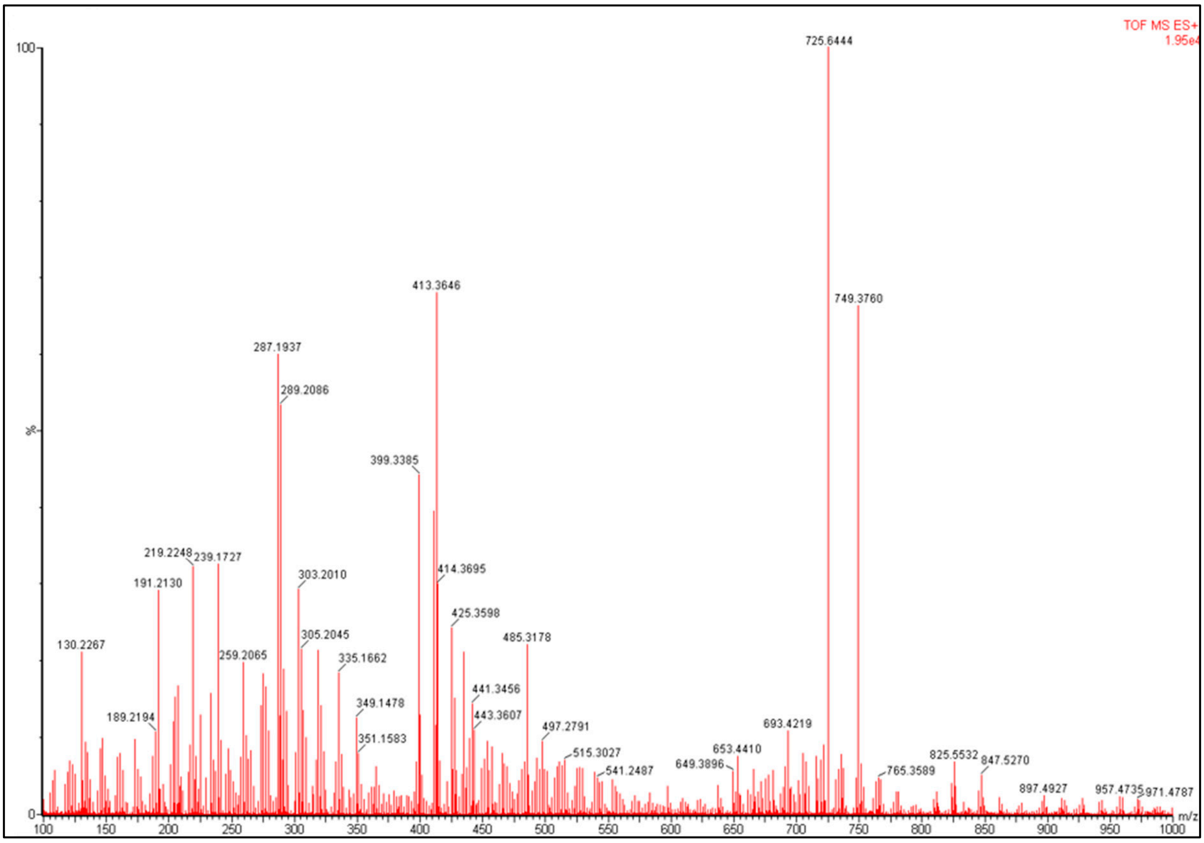

Figure S11. FTIR Spectrum of **2**.

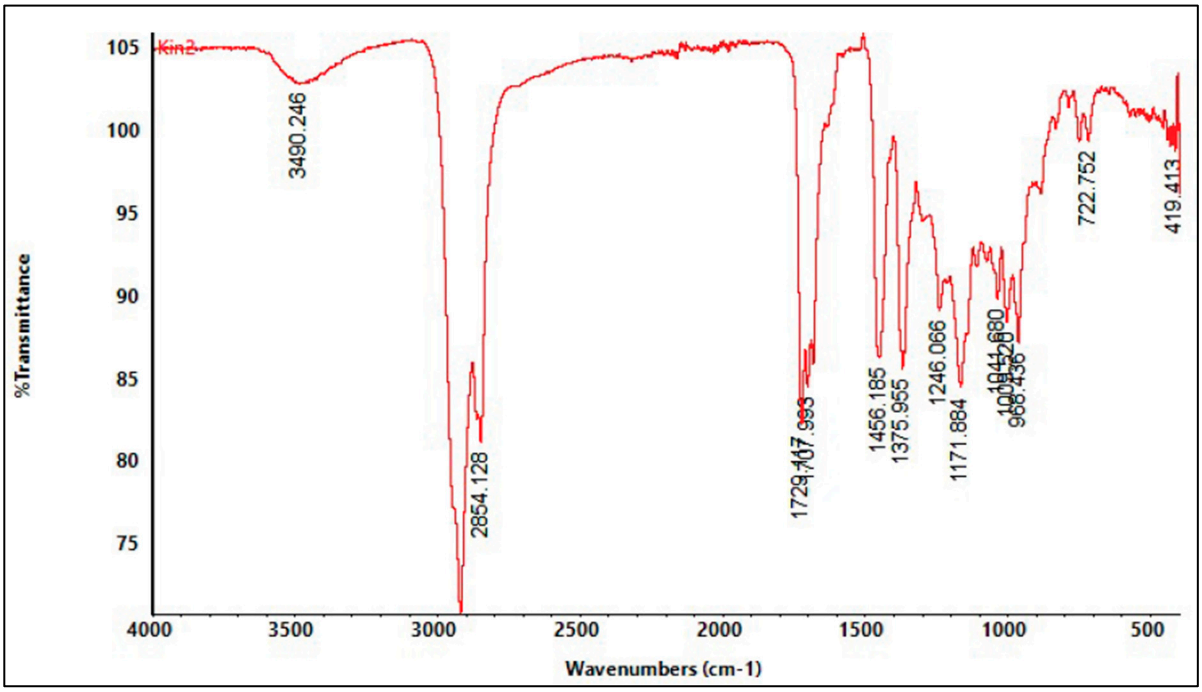

Figure S12. ^{1}H-NMR Spectrum of **2** (500 MHz in $CDCl_3$).

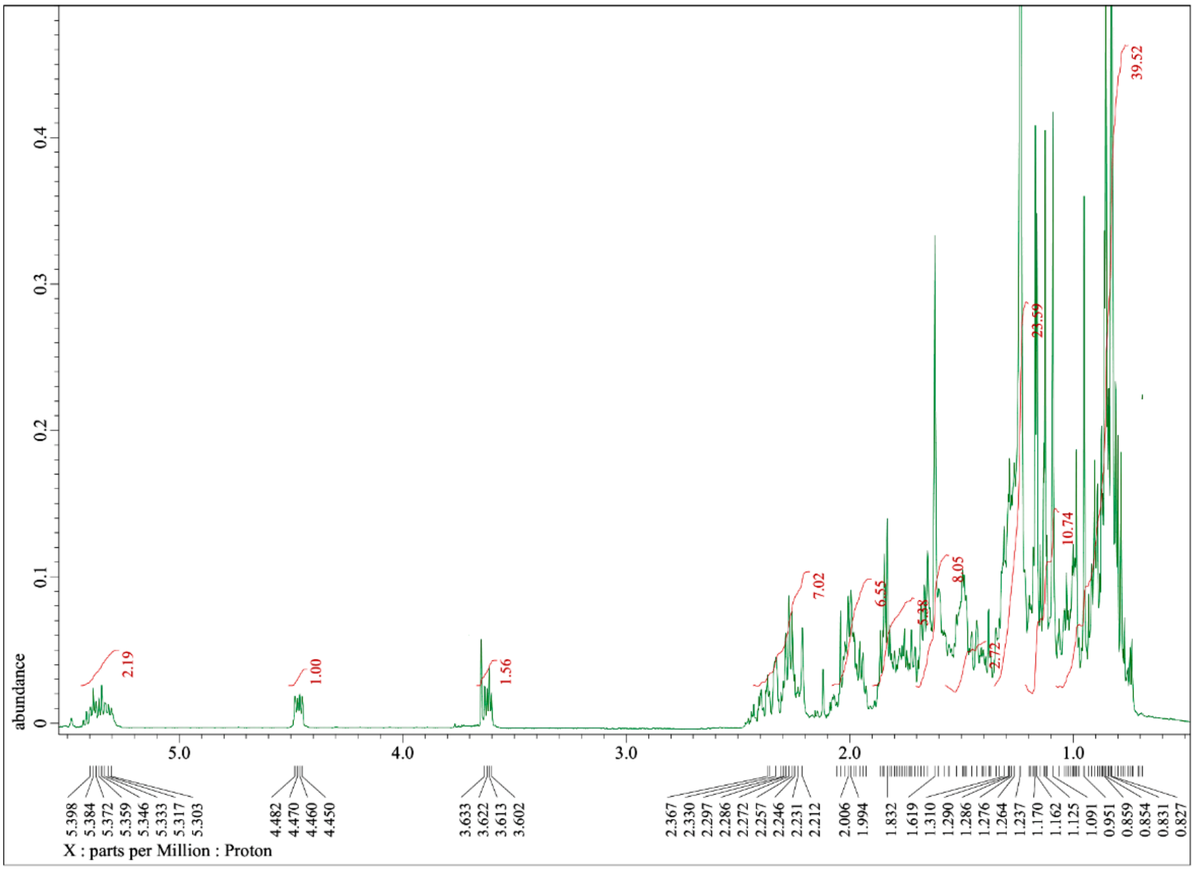

Figure S13. ^{13}C-NMR Spectrum of **2** (125 MHz in $CDCl_3$).

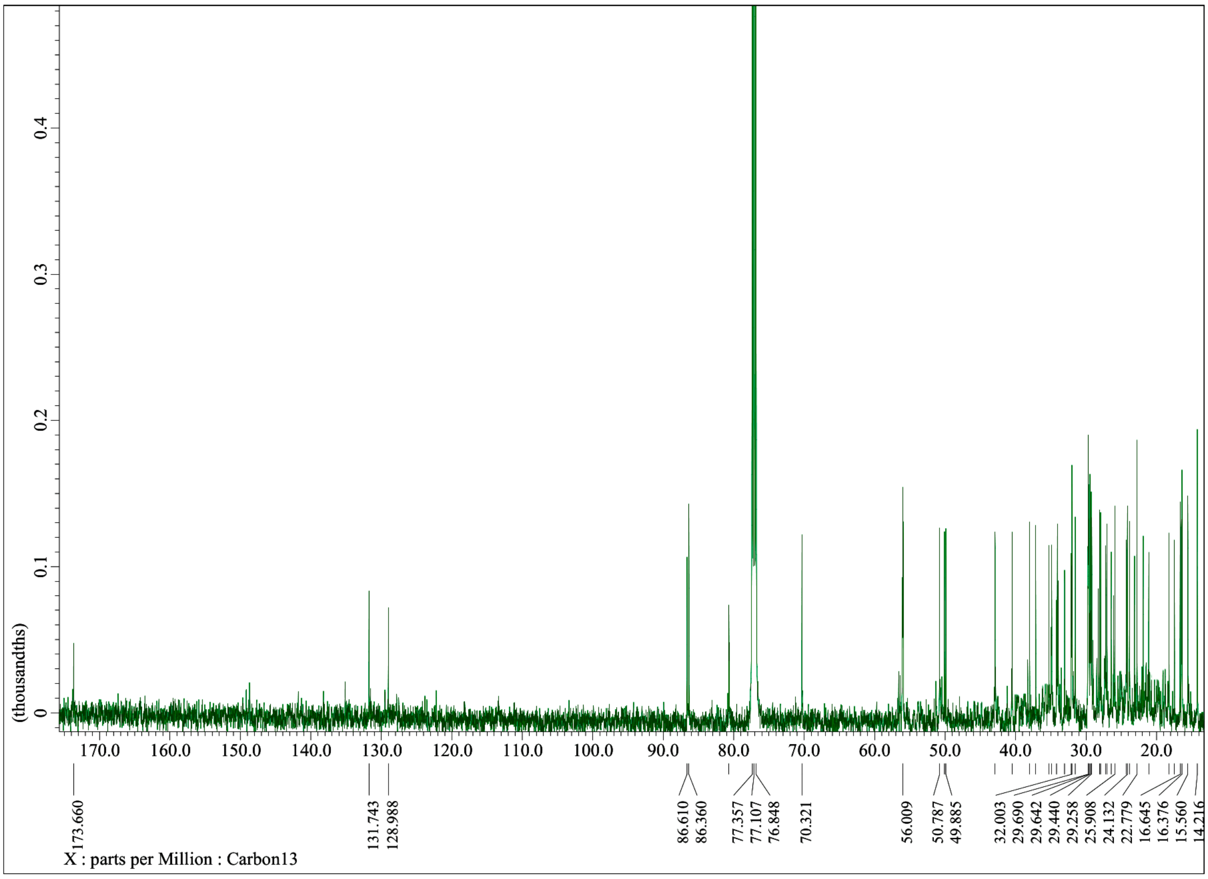

Figure S14. DEPT-135° Spectrum of **2** (125 MHz in $CDCl_3$).

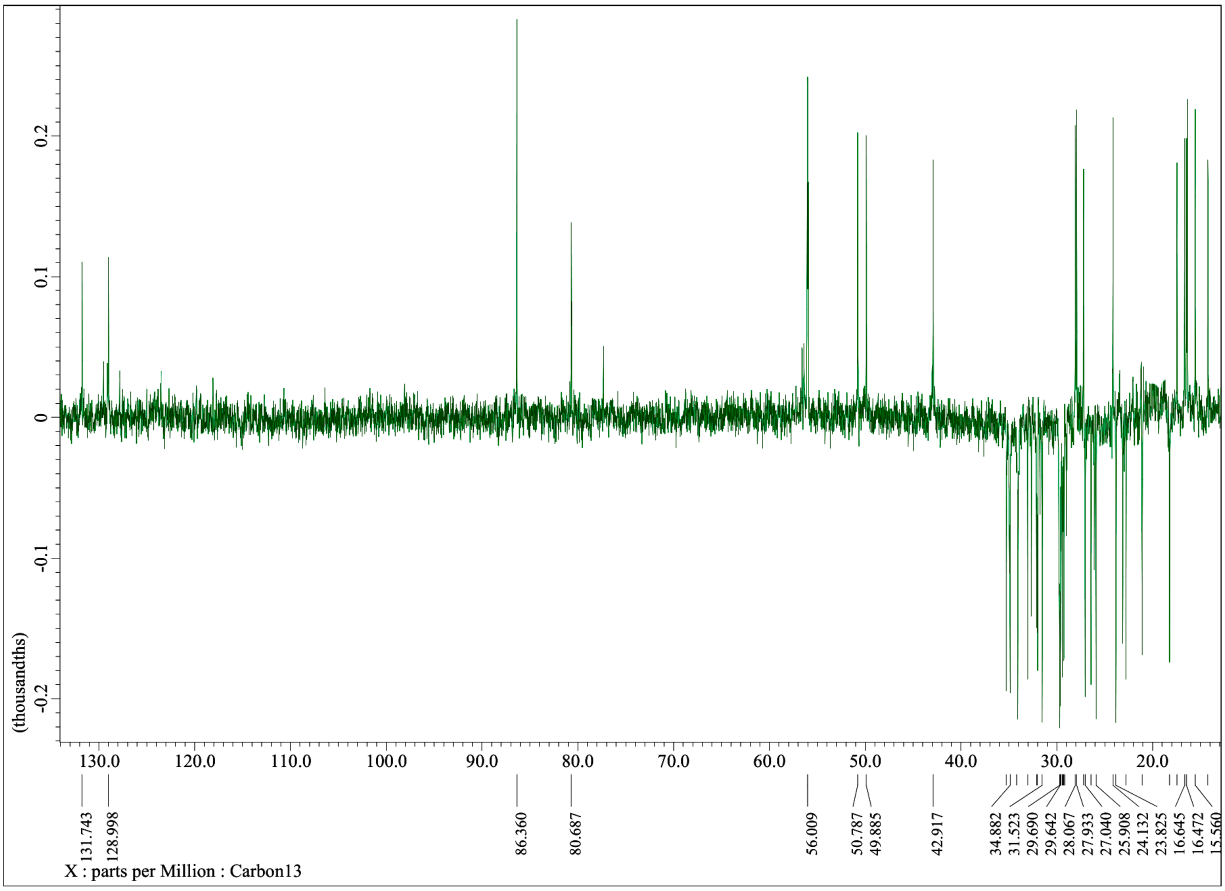

Figure S15. HMQC Spectrum of **2**.

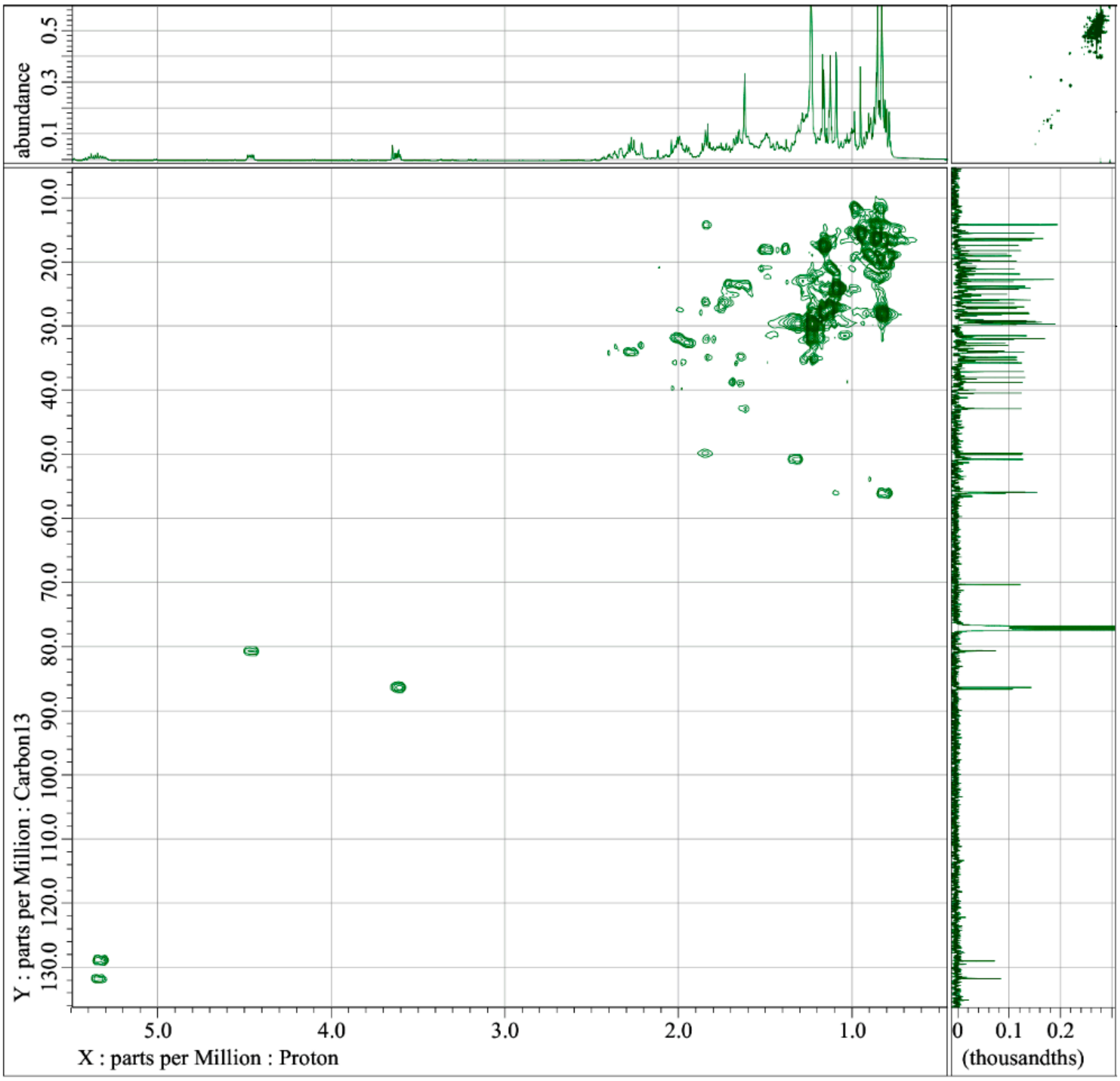

Figure S16. HMBC Spectrum of **2**.

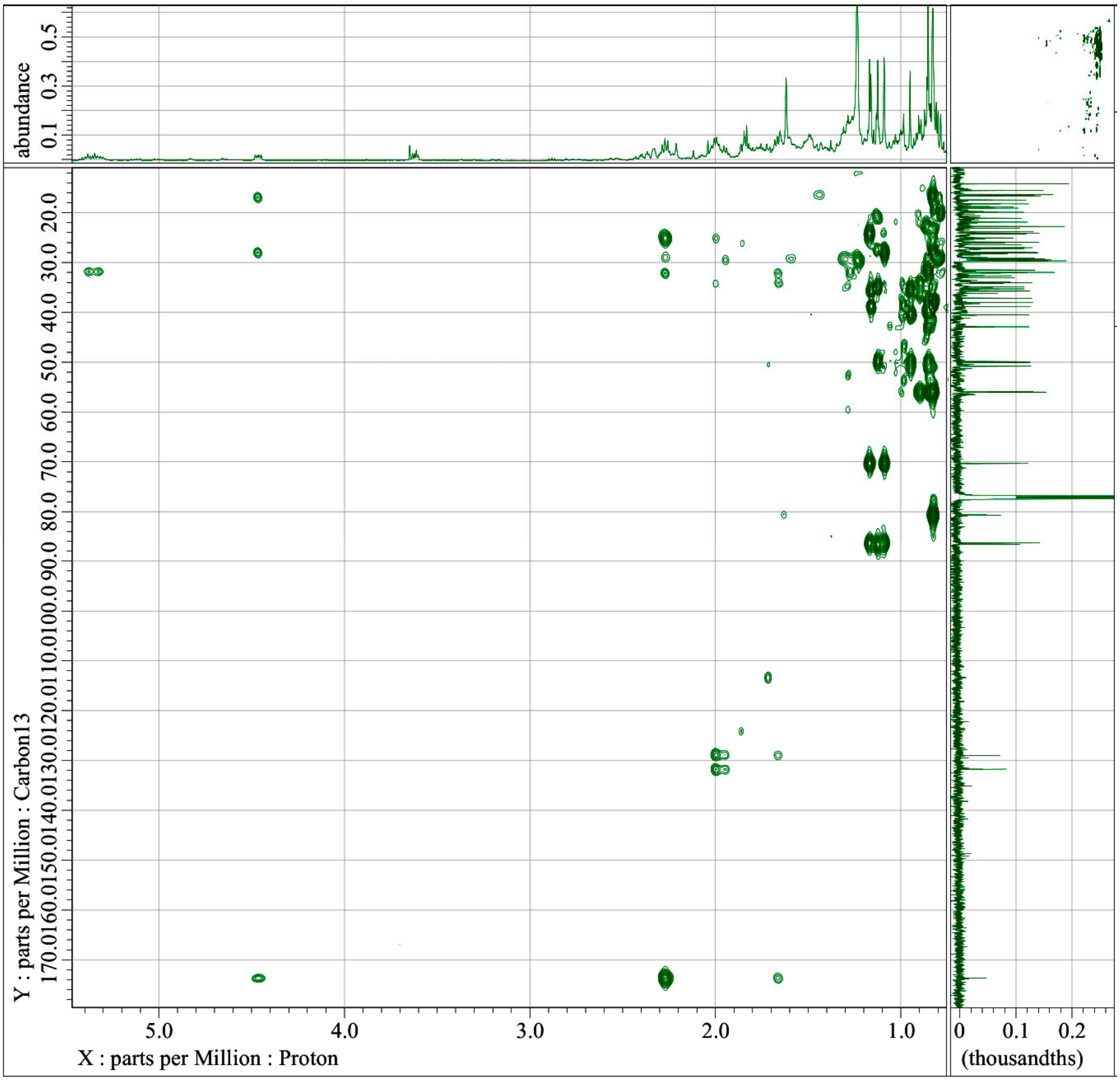

Figure S17. ^{1}H-^{1}H-COSY Spectrum of **2**.

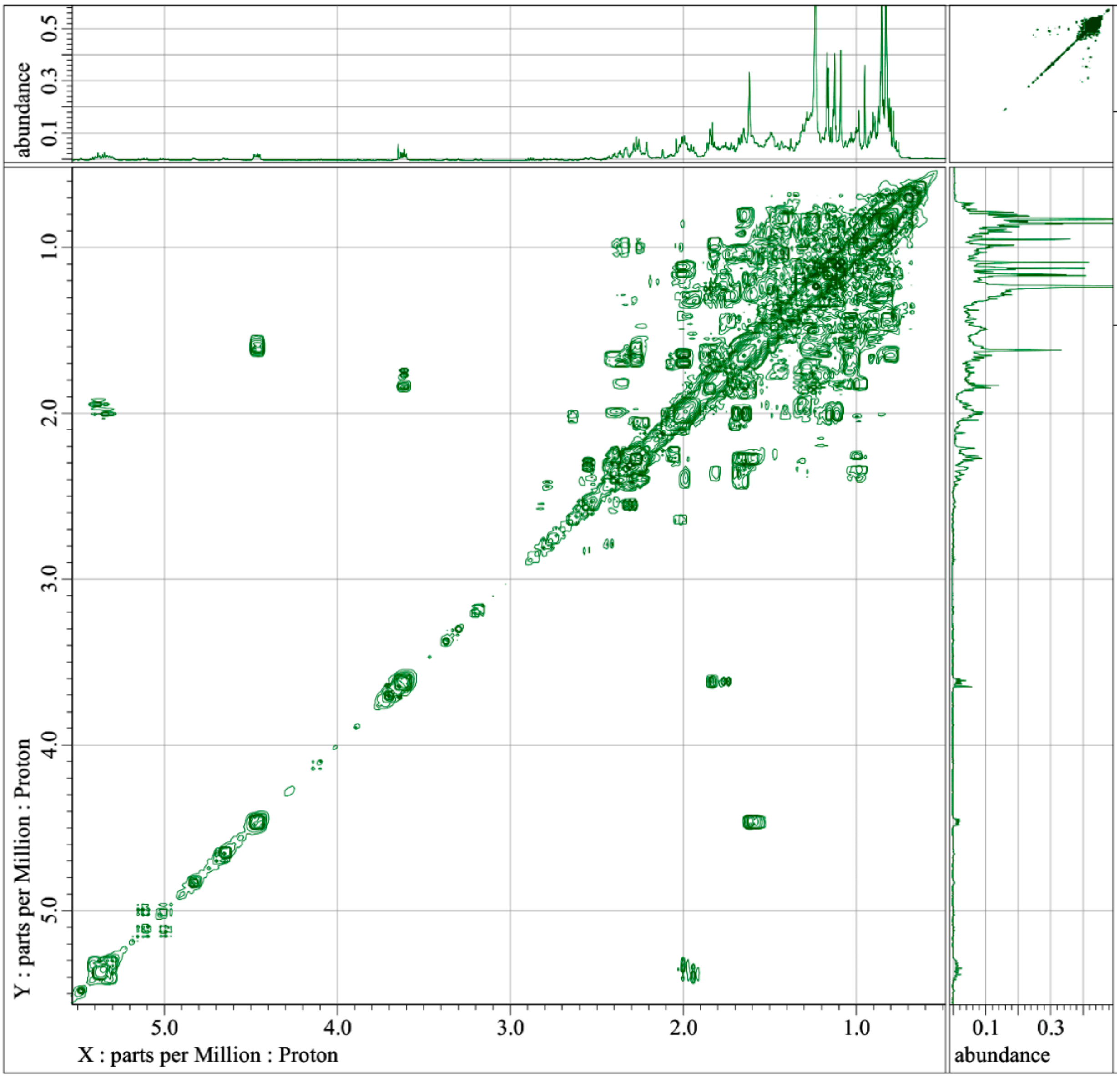

Figure S18. NOESY Spectrum of **2**.

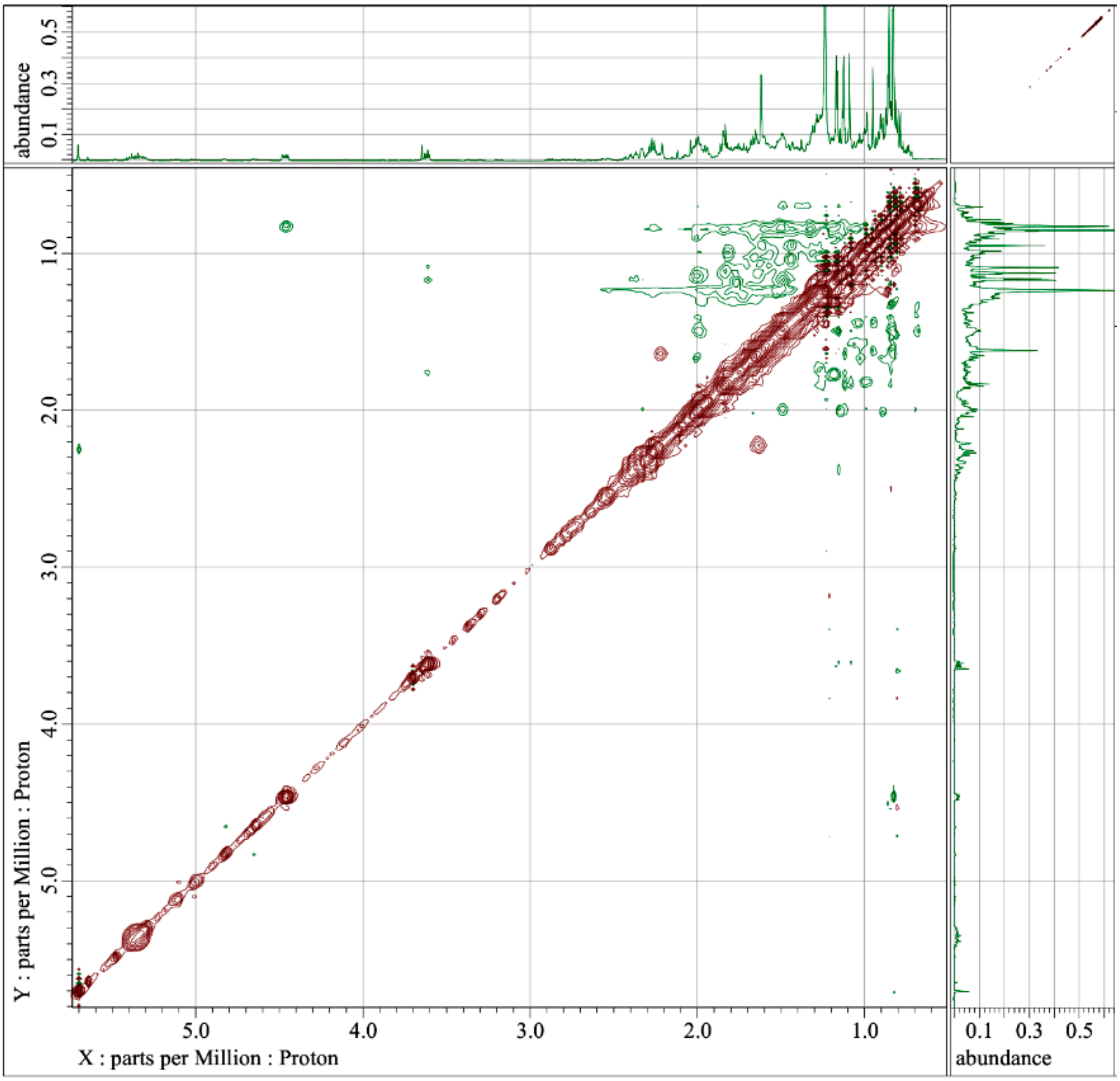

Figure S19. Results of cytotoxic activity of **1** against MCF-7 cell line.

Media	Media+Cell	Cisplatin	dmso	Sample Concentrations (µg/ml)							
			2,00%	500,00	250,00	125,00	62,50	31,25	15,63	7,81	3,91

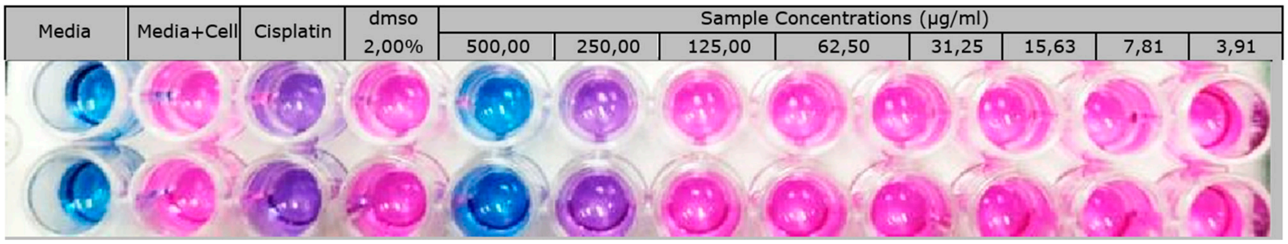

Concentration	Corrected Absorbance	Absorbance Target IC50	Sample Concentration Absorbance IC50
500,00	0,0239	0,3504	313,23
250,00	0,3991		
125,00	0,7034		
62,50	0,7151		
31,25	0,7269		
15,63	0,7269		
7,81	0,7204		
3,91	0,7220		

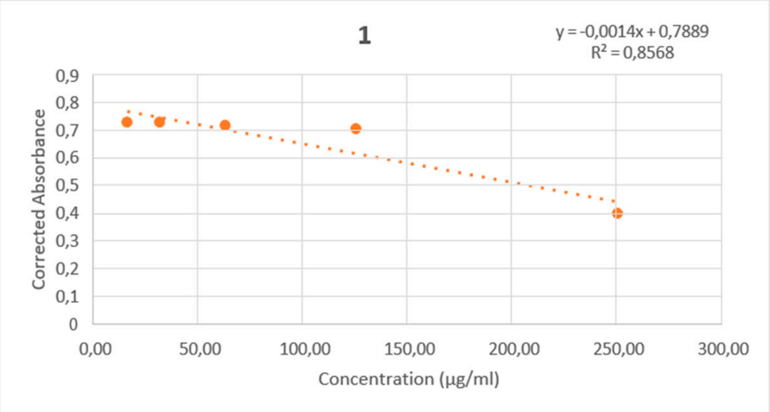

Figure S20. Results of cytotoxic activity of **2** against MCF-7 cell line.

Media	Media+Cell	Cisplatin	dmso	Sample Concentrations (µg/ml)							
			2,00%	500,00	250,00	125,00	62,50	31,25	15,63	7,81	3,91

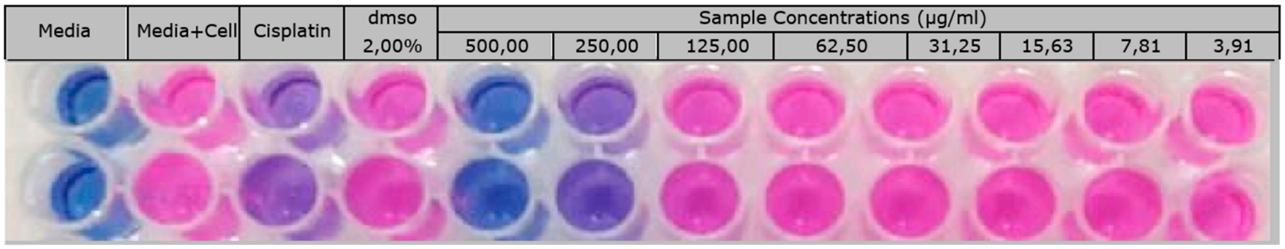

Concentration	Corrected Absorbance	Absorbance Target IC50	Sample Concentration Absorbance IC50
500,00	0,0208	0,3708	212,21
250,00	0,2273		
125,00	0,7015		
62,50	0,7500		
31,25	0,7791		
15,63	0,7891		
7,81	0,7950		
3,91	0,7954		

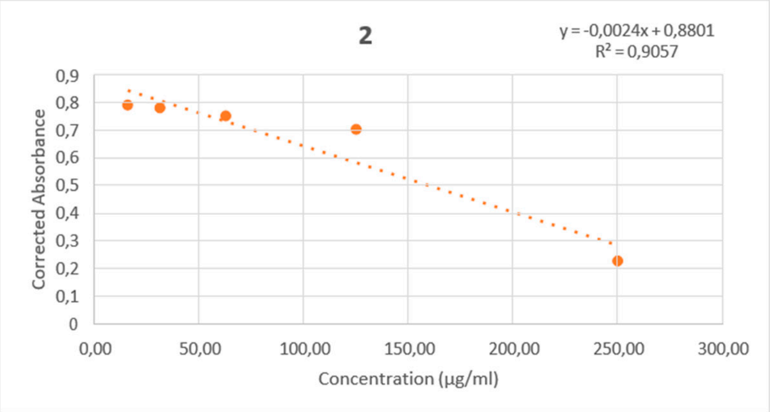

Figure S21. Results of cytotoxic activity of **3** against MCF-7 cell line.

Media	Media+Cell	Cisplatin	dmso	Sample Concentrations (µg/ml)							
			2,00%	500,00	250,00	125,00	62,50	31,25	15,63	7,81	3,91

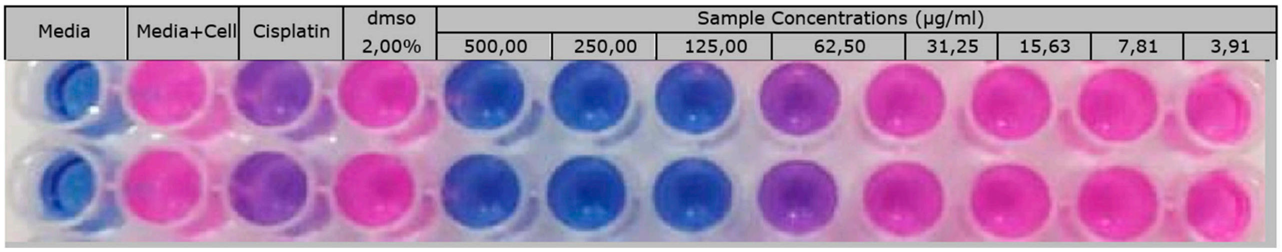

Concentration	Corrected Absorbance
500,00	0,0037
250,00	0,0061
125,00	0,0141
62,50	0,3575
31,25	0,6574
15,63	0,7177
7,81	0,7612
3,91	0,7652

Absorbance Target IC50	Sample Concentration Absorbance IC50
0,3756	67,30

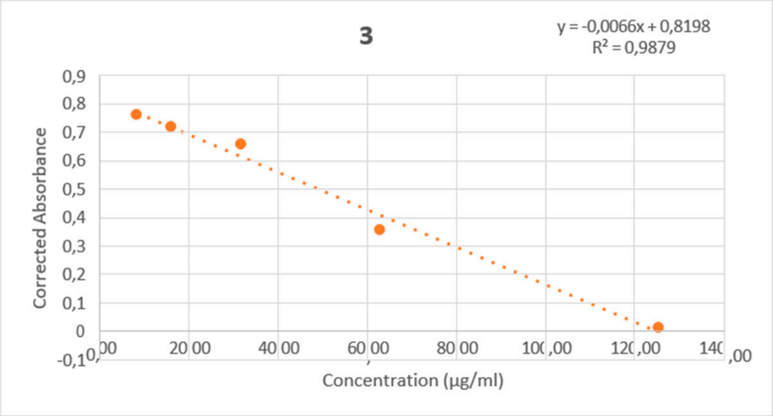

Figure S22. Results of cytotoxic activity of **4** against MCF-7 cell line.

Media	Media+Cell	Cisplatin	dmso	Sample Concentrations (µg/ml)							
			2,00%	500,00	250,00	125,00	62,50	31,25	15,63	7,81	3,91

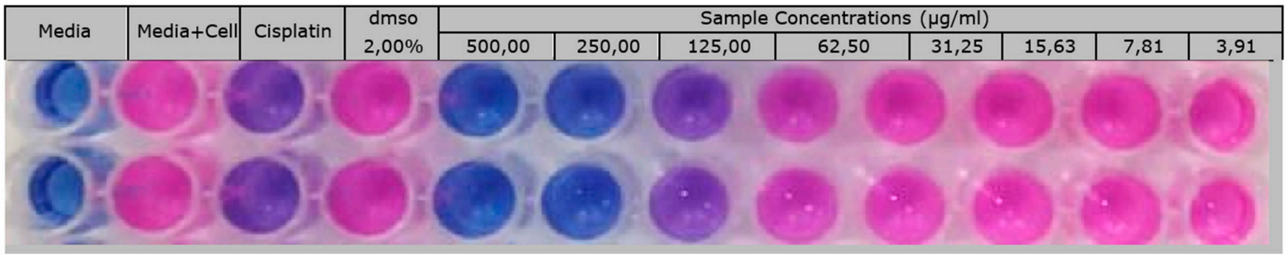

Concentration	Corrected Absorbance
500,00	0,0075
250,00	0,0422
125,00	0,3153
62,50	0,6464
31,25	0,7176
15,63	0,7529
7,81	0,7628
3,91	0,7638

Absorbance Target IC50	Sample Concentration Absorbance IC50
0,3647	121,01

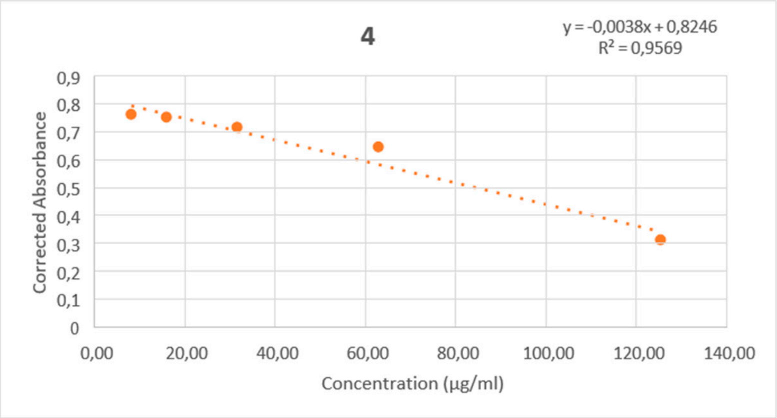

Figure S23. Results of cytotoxic activity of **5** against MCF-7 cell line.

Media	Media+Cell	Cisplatin	dmso	Sample Concentrations (µg/ml)							
			2,00%	250,00	125,00	62,50	31,25	15,63	7,81	3,91	1,95

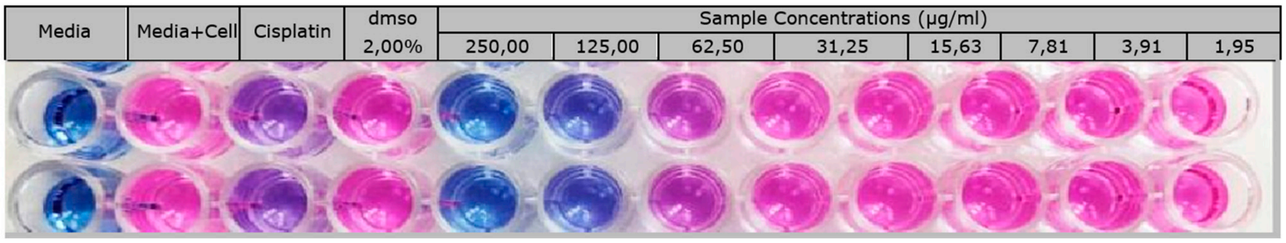

Concentration	Corrected Absorbance	Absorbance Target IC50	Sample Concentration Absorbance IC50
250,00	0,0050	0,3705	82,61
125,00	0,0990		
62,50	0,5271		
31,25	0,6906		
15,63	0,7410		
7,81	0,7604		
3,91	0,7824		
1,95	0,7801		

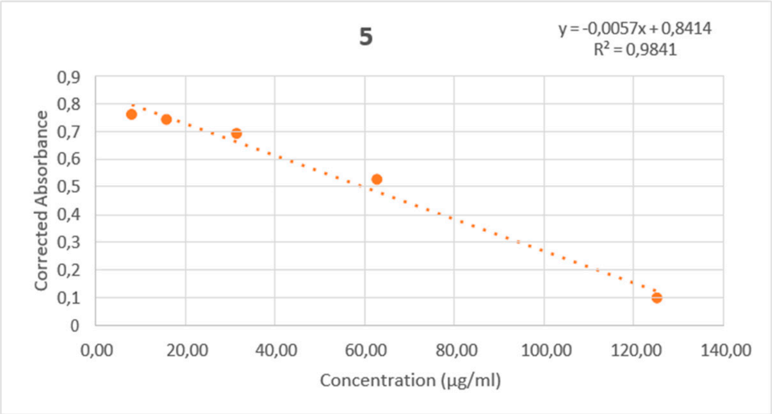

Figure S24. Results of cytotoxic activity of **1** against B16-F10 cell line.

Media	Media+Cell	Cisplatin	dmso	Sample Concentrations (µg/ml)							
			2,00%	1000,00	500,00	250,00	125,00	62,50	31,25	15,63	7,81

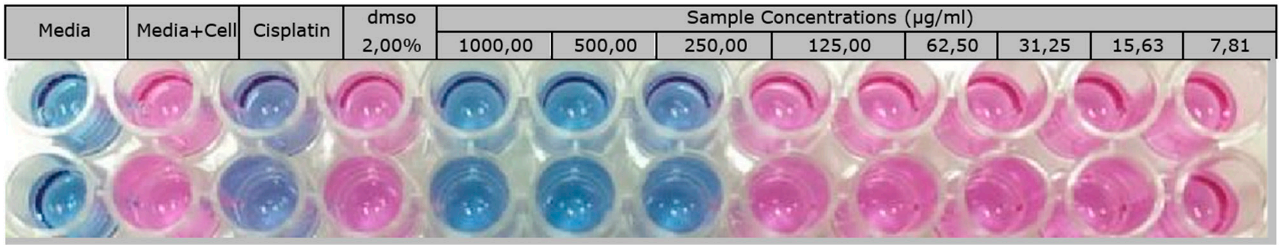

Concentration	Corrected Absorbance	Absorbance Target IC50	Sample Concentration Absorbance IC50
1000,00	0,0089	0,3127	181,34
500,00	0,0090		
250,00	0,0510		
125,00	0,6443		
62,50	0,6709		
31,25	0,6758		
15,63	0,6696		
7,81	0,6802		

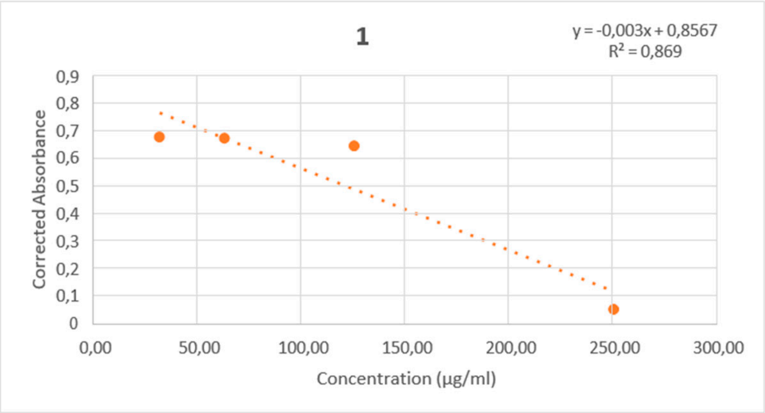

Figure S25. Results of cytotoxic activity of **2** against B16-F10 cell line.

Media	Media+Cell	Cisplatin	dmso 2,00%	Sample Concentrations (µg/ml)							
				500,00	250,00	125,00	62,50	31,25	15,63	7,81	3,91

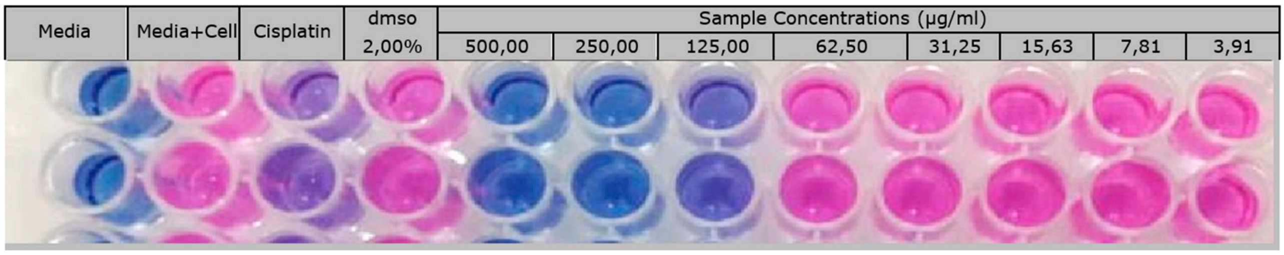

Concentration	Corrected Absorbance
500,00	0,0161
250,00	0,0291
125,00	0,1351
62,50	0,6909
31,25	0,7092
15,63	0,7264
7,81	0,7442
3,91	0,7473

Absorbance Target IC50	Sample Concentration Absorbance IC50
0,3492	98,40

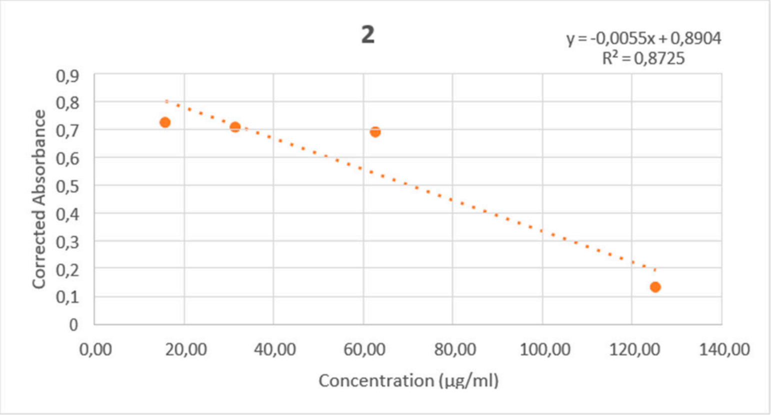

Figure S26. Results of cytotoxic activity of **3** against B16-F10 cell line.

Media	Media+Cell	Cisplatin	dmso 2,00%	Sample Concentrations (µg/ml)							
				500,00	250,00	125,00	62,50	31,25	15,63	7,81	3,91

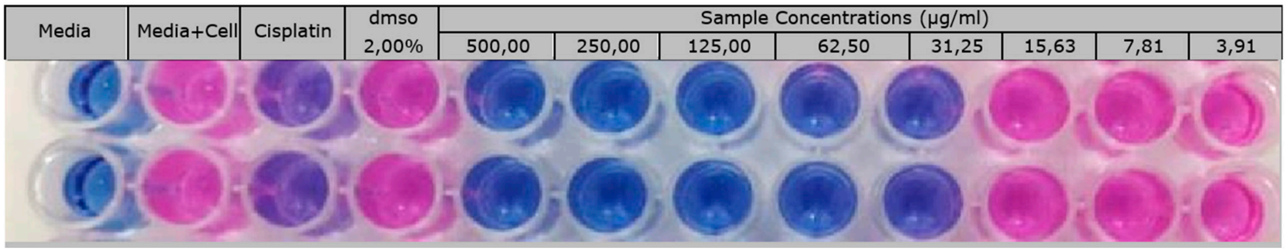

Concentration	Corrected Absorbance
500,00	0,0051
250,00	0,0067
125,00	0,0077
62,50	0,0375
31,25	0,0771
15,63	0,6766
7,81	0,7031
3,91	0,7063

Absorbance Target IC50	Sample Absorbance Concentration IC50
0,3436	22,95

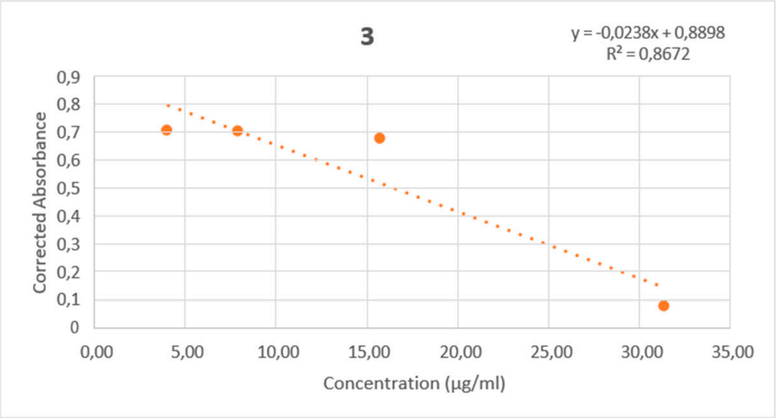

Figure S27. Results of cytotoxic activity of **4** against B16-F10 cell line.

Media	Media+Sel	Cisplatin	dmso 2,00%	Sample Concentration (µg/ml)							
				500,00	250,00	125,00	62,50	31,25	15,63	7,81	3,91

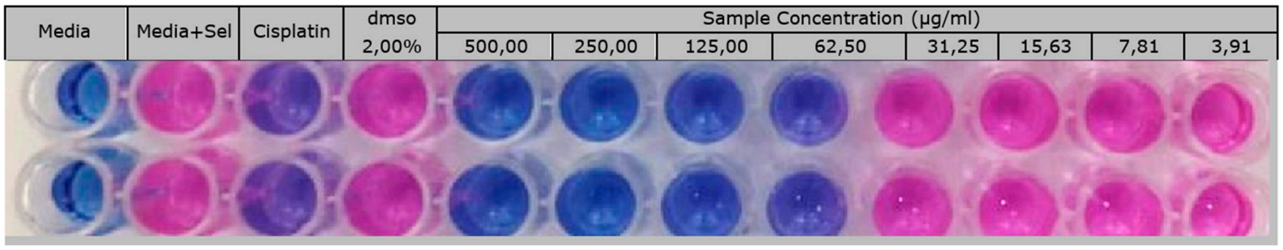

Concentration	Corrected Absorbance	Absorbance Target IC50	Sample Concentration Absorbance IC50
500,00	0,0112	0,3378	49,57
250,00	0,0139		
125,00	0,0584		
62,50	0,1255		
31,25	0,6914		
15,63	0,7163		
7,81	0,7090		
3,91	0,7164		

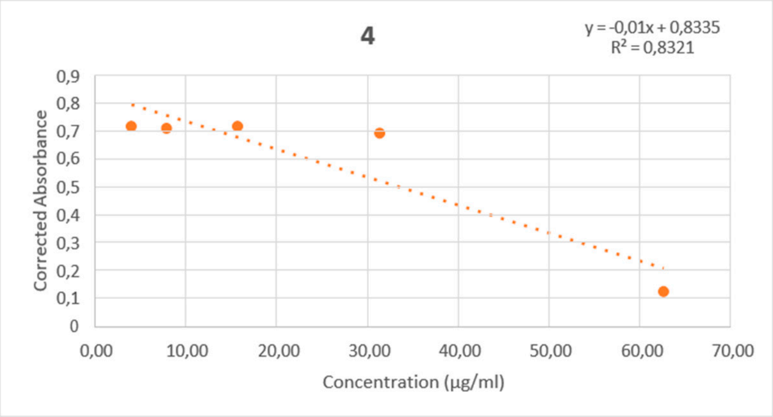

Figure S28. Results of cytotoxic activity of **5** against B16-F10 cell line.

Media	Media+Sel	Cisplatin	dmso 2,00%	Konsentrasi Sampel (µg/ml)							
				250,00	125,00	62,50	31,25	15,63	7,81	3,91	1,95

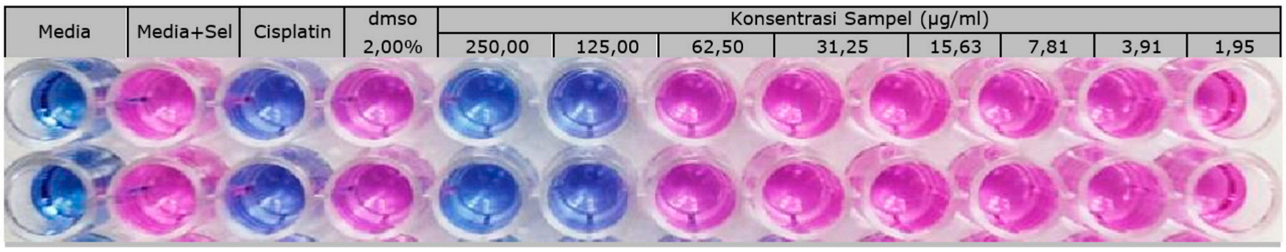

Concentration	Corrected Absorbance	Absorbance Target IC50	Sample Concentration Absorbance IC50
250,00	-0,0011	0,2764	95,27
125,00	0,0657		
62,50	0,5589		
31,25	0,6090		
15,63	0,6383		
7,81	0,6211		
3,91	0,6243		
1,95	0,6320		

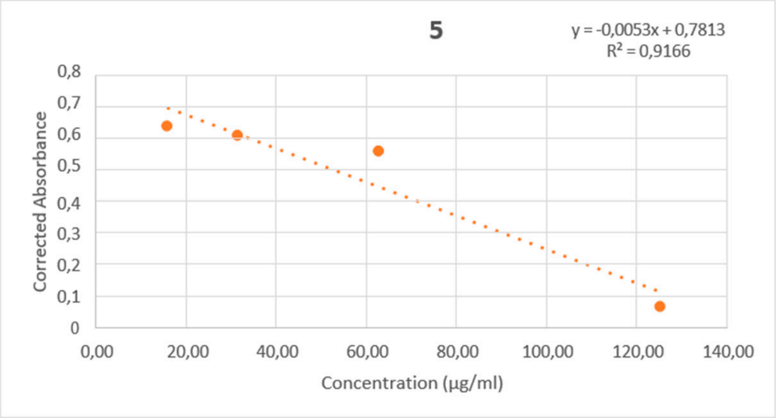

Supplement: Supplementary file 1 [file molecules-27-06757-s001.zip › molecules-1947175-supplementary.pdf]
